# Multimodal microscopy for automated histologic analysis of prostate cancer

**DOI:** 10.1186/1471-2407-11-62

**Published:** 2011-02-09

**Authors:** Jin Tae Kwak, Stephen M Hewitt, Saurabh Sinha, Rohit Bhargava

**Affiliations:** 1 Department of Computer Science, University of Illinois at Urbana-Champaign, Urbana, IL 61801, USA; 2 Beckman Institute for Advanced Science and Technology, University of Illinois at Urbana-Champaign, Urbana, IL 61801, USA; 3 Tissue array research program, National Cancer Institute, National Institutes of Health, Bethesda, MD 20850, USA; 4 Department of Bioengineering, University of Illinois at Urbana-Champaign, Urbana, IL 61801, USA

## Abstract

**Background:**

Prostate cancer is the single most prevalent cancer in US men whose gold standard of diagnosis is histologic assessment of biopsies. Manual assessment of stained tissue of all biopsies limits speed and accuracy in clinical practice and research of prostate cancer diagnosis. We sought to develop a fully-automated multimodal microscopy method to distinguish cancerous from non-cancerous tissue samples.

**Methods:**

We recorded chemical data from an unstained tissue microarray (TMA) using Fourier transform infrared (FT-IR) spectroscopic imaging. Using pattern recognition, we identified epithelial cells without user input. We fused the cell type information with the corresponding stained images commonly used in clinical practice. Extracted morphological features, optimized by two-stage feature selection method using a minimum-redundancy-maximal-relevance (mRMR) criterion and sequential floating forward selection (SFFS), were applied to classify tissue samples as cancer or non-cancer.

**Results:**

We achieved high accuracy (area under ROC curve (AUC) >0.97) in cross-validations on each of two data sets that were stained under different conditions. When the classifier was trained on one data set and tested on the other data set, an AUC value of ~0.95 was observed. In the absence of IR data, the performance of the same classification system dropped for both data sets and between data sets.

**Conclusions:**

We were able to achieve very effective fusion of the information from two different images that provide very different types of data with different characteristics. The method is entirely transparent to a user and does not involve any adjustment or decision-making based on spectral data. By combining the IR and optical data, we achieved high accurate classification.

## Background

### Prostate cancer

Prostate cancer (PCa) is the single most prevalent cancer in US men, accounting for one-third of non-skin cancer diagnoses every year [1]. Screening for the disease is widespread and for almost a million cases a year [2-4], a biopsy is conducted to detect or rule out cancer [3]. Manually-conducted histologic assessment of tissue upon biopsy forms the definitive diagnosis of PCa [5]. This need places a large demand on pathology services and manual examination limits speed and throughput. Histologic assessment is also critical to scientific progress as it is often the basis for research studies. Alternative methods for histologic recognition can greatly aid in alleviating workloads, assuring quality control and reducing costs [6]. There is no straightforward way, however, to aid pathology in this task and no clinical instrument is available for routine use. Hence, high-throughput, automated and objective tools for prostate pathology - both in clinical practice and in research - are needed.

### Optical microscopy and automated PCa detection

**Figure 1:**
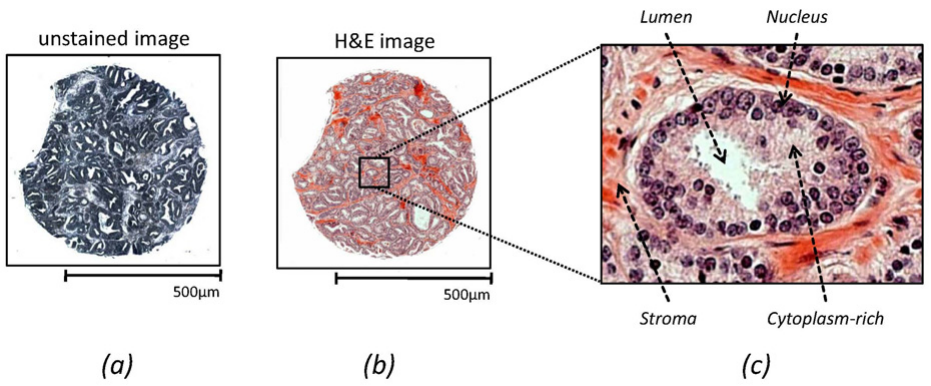
**Staining allows visualization of tissue features**. (a) an unstained image has little contrast while (b) the application of H&E stain highlights nucleic acid-rich regions as blue and protein-rich regions at pink. (c) structure of a prostate gland. It is notable that the stain is universal in that it is not diagnostic of cell type or disease. The stain serves only to provide contrast that is subsequently used by a human to recognize cell types and diagnose disease.

Since the tissue does not have appreciable contrast in optical brightfield microscopy (Figure 1A), tissue samples are commonly stained using hematoxylin and eosin (H&E) prior to review by a pathologist. The stain is specific in limited terms - staining protein-rich regions pink and nucleic acid-rich regions of the tissue blue (Figure 1B). A pathologist is trained to recognize, from a stained tissue sample, the morphology and local architecture of glands as well as their structural alterations that indicate disease. The specific cell type that is used to recognize glandular structures is the epithelial sub-type. In prostatic carcinoma, which comprises more than 95% of prostate cancers [5], the cells of interest are epithelial cells [7]. Epithelial cells line 3D ducts in intact tissue and, hence, appear as cells lining empty circular regions (lumens) in images of histologic sections. Patterns of distortions of lumen appearance and spacing, as well as the arrangement of epithelial cells relative to lumens, have been characterized to indicate cancer and characterize its severity (Gleason grade) [8,9]. The greater the distortion and loss of regular structure, the worse (higher grade) the cancer.

Recognizing structural distortions indicative of disease is a manual pattern recognition process that matches patterns in the tissue sample to standard patterns. Manual examination is powerful in that humans can recognize disease from a wide spectrum of normal and disease states, can overcome confounding artifacts, detect unusual cases and even recognize deficiencies in diagnoses. Manual examination, unfortunately, is time-consuming and leads routinely to variability in grading disease [8]. Computer-aided recognition of disease samples and grade patterns [10], hence, holds the potential for more accurate, reproducible and automated diagnoses [11,12]. Unfortunately, tissue samples stain variably in populations due to biological diversity, with variations in stain composition, processing conditions and histotechnologists. The net result confounds automated image analysis and human-competitive recognition of cancer has not been automated for routine use. A robust means of automatically detecting epithelium and correlating its spatial patterns to determining cancer presence is highly desirable but yet unsolved.

Several efforts have been made to develop automated systems for the diagnosis and grading of microscopic prostate images. These include methods to identify distinct tissue compositions [13,14] as well as several methods for automatic grading [15-23]. The majority of these methods have extracted texture and/or morphological features to characterize tissue samples. Histologic objects such as nuclei, lumen, or gland have been mainly used to extract morphological features [15,16,20-22,24,25]. Fourier Transform [17], Wavelet Transform [18,19,22], and Fractal Analysis [22,23] have been the techniques commonly used to obtain texture features. In addition to these features, color [22] and graph-based [20] features have also been used. A number of classifiers have been tested on various features and data sets, although the choice of classifiers seems to have been less significant than the feature extraction step [22,23].

Despite the above-mentioned lines of progress in automated diagnosis, an important concern is that the varying properties of images, due to acquisition settings [19,25] and staining [26], may affect the classification results substantially. Although the issue of image variation by different acquisition settings has been addressed in [19,25], to the best of our knowledge, no previous method has been validated across data sets under different staining conditions.

A major roadblock has been the limited information present in the data. For example, different cell types and morphologies are recognized by recognizing colors for empty space (usually close to white), apical portion of epithelial cells (usually pink) and the basal layer of epithelial cells (usually pink-dark blue). Immunohistochemical probes add useful information to diagnostic processes and are effective in understanding specific aspects of the disease, e.g. loss of basement membrane. For routine diagnostic pathology, however, the use of such molecular stains is expensive, time-consuming and does not actually address the need for an operator-free method. Additional molecular data is now available using label-free spectroscopic imaging, also known as chemical imaging [27].

### Chemical imaging and automated histologic classification

**Figure 2:**
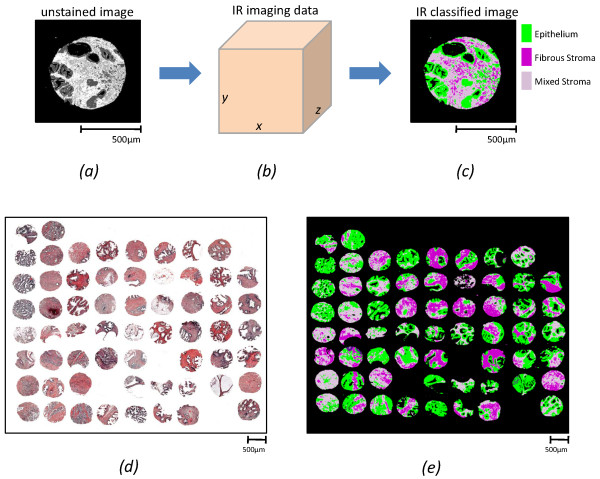
**IR imaging data and its use in histologic classification**. (Upper row) IR imaging data (b) is acquired for an unstained tissue section (a). The data is then classified into cell types and a classified image (c) is obtained. The colors indicate cell types in a histologic model of prostate tissue. This method is robust and applied to hundreds of tissue samples using the tissue microarray (TMA) format. (Lower row) H&E (d) and IR classified (e) images of a part of the TMAs used.

Prostatic epithelial cells (and other cell types) [28] have recently been automatically recognized using a novel form of chemical imaging based on mid-infrared (IR) spectroscopy. Fourier transform infrared (FT-IR) spectroscopic imaging provides non-perturbing imaging by combining the spatial specificity of optical microscopy with the molecular selectivity of vibrational spectroscopy. Mid-IR spectral frequencies are resonant with the fundamental vibrational mode frequencies in molecules; hence, the IR absorption spectrum at each pixel is a quantitative record of composition [29]. FT-IR imaging has been successfully applied to various biological and biomedical problems such as determining molecular concentrations [30,31] and structure [32,33], characterizing cell components [34] and cancer diagnosis [35-37]. In particular, the spectral patterns of different cell types being different, computerized pattern recognition can be used to assign each pixel into constituent cell types. The final result of recording data and mathematical analysis is images of tissue that are color coded for cell type. The process is illustrated in Figure 2. The approach has been used by a number of groups and is summarized in recent edited volumes [38,39]. Since the numerical algorithms are automated, quantification of accuracy and statistical confidence in results is facile [40].

The above approach has been extensively validated in providing histologic recognition using tissue samples from over 1000 patients and tens of millions of pixels using tissue microarrays (TMAs). TMAs consist of multiple tissue samples of a size that assures representative sampling and allow high throughput experimentation in an efficient manner. For this manuscript, we examined two independent data sets from prostate tissue microarrays that were subjected to chemical imaging and histologic classification as outlined above. Images of the data are shown in Figure 2.

While we expected the chemical imaging approach to prove useful in histologic analysis of prostate tissue, its relationship to the existing clinical practice of using H&E stained tissue in PCa diagnosis was not clear *a priori*. Hence, we sought to examine whether a combination of the two techniques (i.e., optical microscopy following H&E staining, and FT-IR imaging) could provide high accuracy diagnoses that could otherwise not be achieved using H&E images alone.

### Overview of this work

We develop a new fully-automated method to classify cancer versus non-cancer prostate tissue samples. The classification algorithm uses morphological features - geometric properties of epithelial cells/nuclei and lumens - that are quantified based on H&E stained images as well as FT-IR images of the tissue samples. By restricting the features used to geometric measures, we sought to mimic the pattern recognition process employed by human experts, and achieve a robust classification procedure that can produce consistently high accuracy across independent data sets. We systematically evaluate the performance of the new method through cross-validation, and examine its robustness across data sets. We also summarize the specific morphological features that prove to be most informative in classification.

## Methods

**Figure 3:**
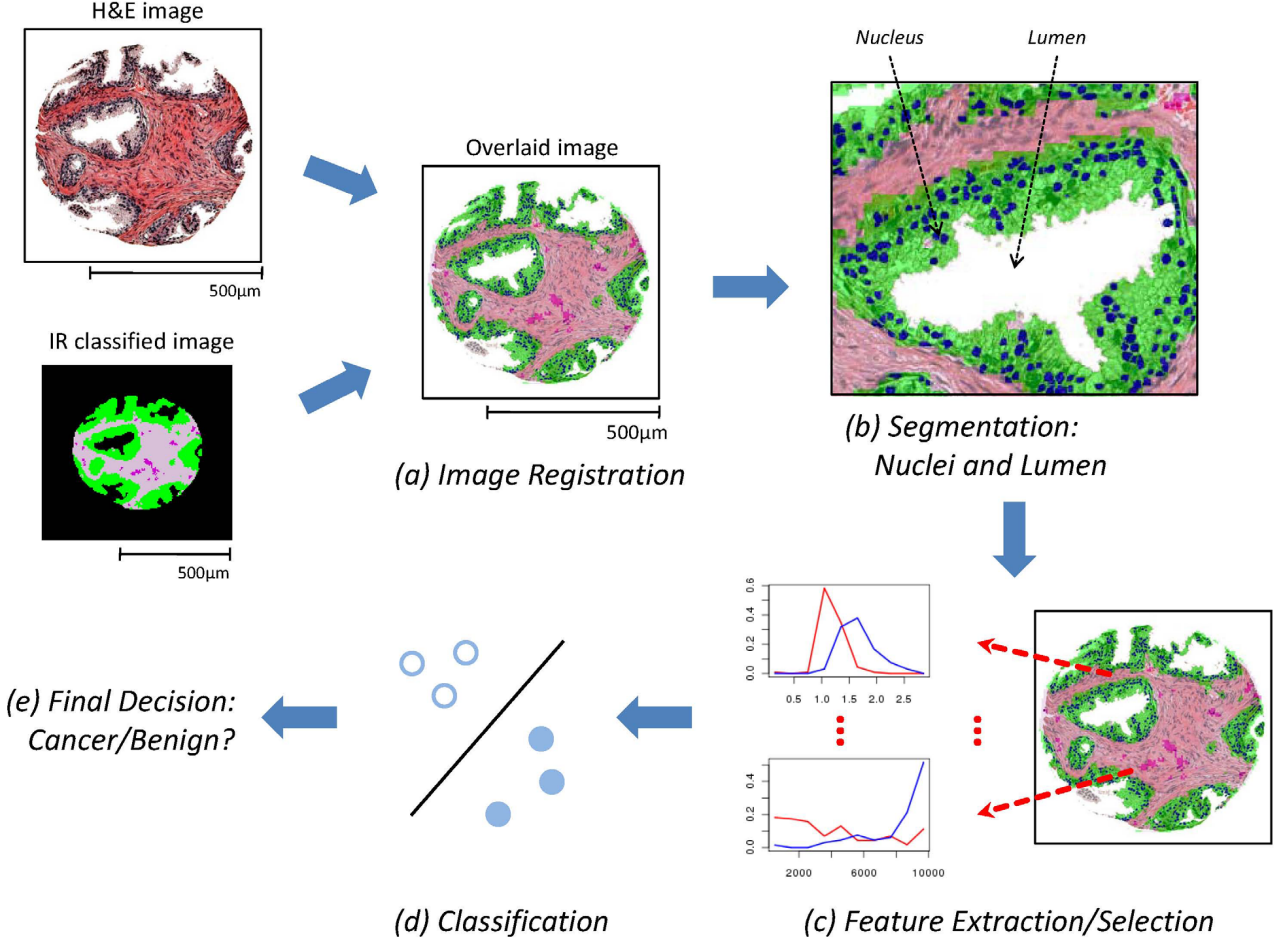
**Overview of System**. (a, b) FTIR spectroscopic imaging data-based cell-type classification (IR classified image), is overlaid with H&E stained image (a), leading to segmentation of nuclei and lumens in a tissue sample (b). (c,d,e) Features are extracted and selected (c), and used by the classifier (d) to predict (e) whether the sample is cancerous or benign.

We begin with a description of the computational pipeline. As noted above, a key aspect of our approach is the use of FT-IR imaging data on a serial section that is H&E-stained to enhance the segmentation of nuclei and lumens. The first two components of the pipeline are geared to this functionality, while the next three components exploit the segmented features obtained from image data to classify the tissue sample (Figure 3).

### Image Registration

Given two images, the image registration problem can be defined as finding the optimal spatial and intensity transformation [41] of one image to the other. Here, two images are H&E stained (*I_reference_*) and "IR classified" images (*I_target_*) which were acquired from adjacent tissue samples. The IR classified image represents the FT-IR imaging data, processed as indicated in Figure 2, to classify each pixel as a particular cell type. Although the two tissue samples were physically in the same intact tissue and are structurally similar, the two images have different properties (total image and pixel sizes, contrast mechanisms and data values). Hence, features to spatially register the images are not trivial. The H&E image provides detailed morphological information that could ordinarily be used for registration, but the IR image lacks such information. On the other hand, the IR image specifies the exact areas corresponding to each cell type, but the difficulty in precisely extracting such regions from the H&E image hinders us from using cell-type information for registration. The only obvious features are macroscopic tissue sample shape and empty space (lumens) inside the tissue samples. To utilize these two features and to avoid problems due to differences in the two imaging techniques, both images are first converted into binary images. Due to the binarization, the intensity transformation is not necessary. As a spatial transformation, we use an affine transformation ( *f *) [41] where a coordinate (*x_1_*, *y_1_*) is transformed to the (*x_2_*, *y_2_*) coordinate after translations (*t_x_*, *t_y_*), rotation by *θ*, and scaling by factor *s*.

$$\left[\begin{array}{l} x_{2}\\ y_{2} \end{array}\right]=\left[\begin{array}{l} t_{x}\\ t_{y} \end{array}\right]+s\left[\begin{array}{cc} \cos\theta & -\sin\theta \\ \sin\theta & \cos\theta \end{array}\right]\left[\begin{array}{l} x_{1}\\ y_{1} \end{array}\right]$$

**Figure 4:**
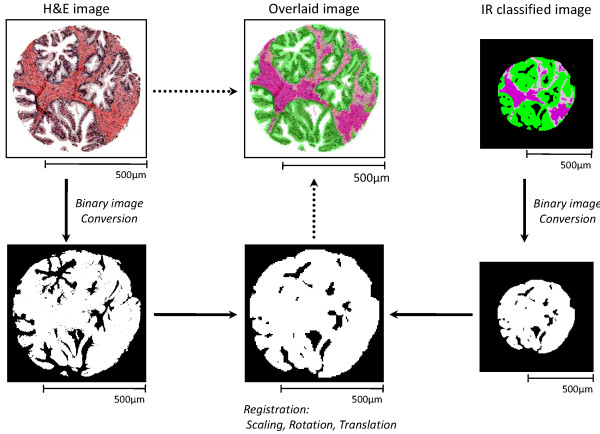
**Image Registration**. H&E stained images and IR classified images are first converted into binary images. The IR classified image is overlaid with the H&E stained image by affine transformation, with the optimal matching being found by minimizing the absolute intensity difference between two images. After registration, original annotations (color and/or cell-type information) of each image are restored.

Accordingly, we find the optimal parameters of the affine transformation that minimizes the absolute intensity difference between two images (*I_reference _*and *I_target_*). In other words, image registration amounts to finding the optimal parameter values $(t_{x}^{*},t_{y}^{*},\theta^{*},s^{*})=\underset{t_{x},t_{y},\theta ,s}{\arg\min}\left|I_{reference}-f(I_{target};t_{x},t_{y},\theta ,s)\right|$. The downhill simplex method [42] is applied to solve the above equation. An example of this registration process is shown in Figure 4. (See [Additional file 1: Image Registration] for details.)

### Identification of epithelial cells and their morphologic features

While a number of factors are known to be transformed in cancerous tissues, epithelial morphology is utilized as the clinical gold standard. Hence, we focus here on cellular and nuclear morphology of epithelial nuclei and lumens. These structures are different in normal and cancerous tissues, but are not widely used in automated analysis due to a few reasons. First, as described above, simple detection of epithelium from H&E images is difficult. Second, detection of epithelial nuclei may be confounded by a stromal response that is not uniform for all grades and types of cancers. We focused first on addressing these two challenges that hinder automatically parsing morphologic features such as the size and number of epithelial nuclei and lumens, distance from nuclei to lumens, geometry of the nuclei and lumens, and others (Feature Extraction). In order to use these properties, the first step is to detect nuclei and lumens correctly and we sought to develop a robust strategy for the same.

#### Lumen Detection

In H&E stained images, lumens are recognized to be empty white spaces surrounded by epithelial cells. In normal tissues, lumens are larger in diameter and can have a variety of shapes. In cancerous tissues, lumens are progressively smaller with increasing grade and generally have less distorted elliptical or circular shapes. Our strategy to detect lumens was to find empty areas that are located next to the areas rich in epithelium. White spots inside the tissue sample can be found from the H&E image by using a proper threshold value (>200) for the intensity of Red, Green, and Blue channels, and the pixels corresponding to epithelial cells can be mapped on the H&E image from the IR classified image through image registration. Although restricting the white areas adjacent to epithelial cells, in our observations, many artifactual lumens are still present. Additionally, the size and shape of lumens are examined to eliminate such artifacts. We note that while lumens are ideally completely surrounded by epithelial cells (called complete lumens), some tissue samples have lumens (called incomplete lumens) that violate this criterion because only a part of lumen is present in the tissue sample. To identify these incomplete lumens, we model an entire tissue sample as a circle, and the white spots between the tissue sample and the circle are the candidate incomplete lumens. As did in complete lumen detection, the same threshold value is used to identify white areas. To identify artifacts, we use heuristic criteria based on the size, shape, presence of epithelial cells and background around the areas. In addition, the distances from the center of the tissue to the white spots are examined to identify the artifacts in crescent form which resulted from the small gaps between the tissue sample and the circle fitted to the sample. (See [Additional file 1: Lumen Detection] for details.)

#### Nucleus Detection - single epithelial cells

**Figure 5:**
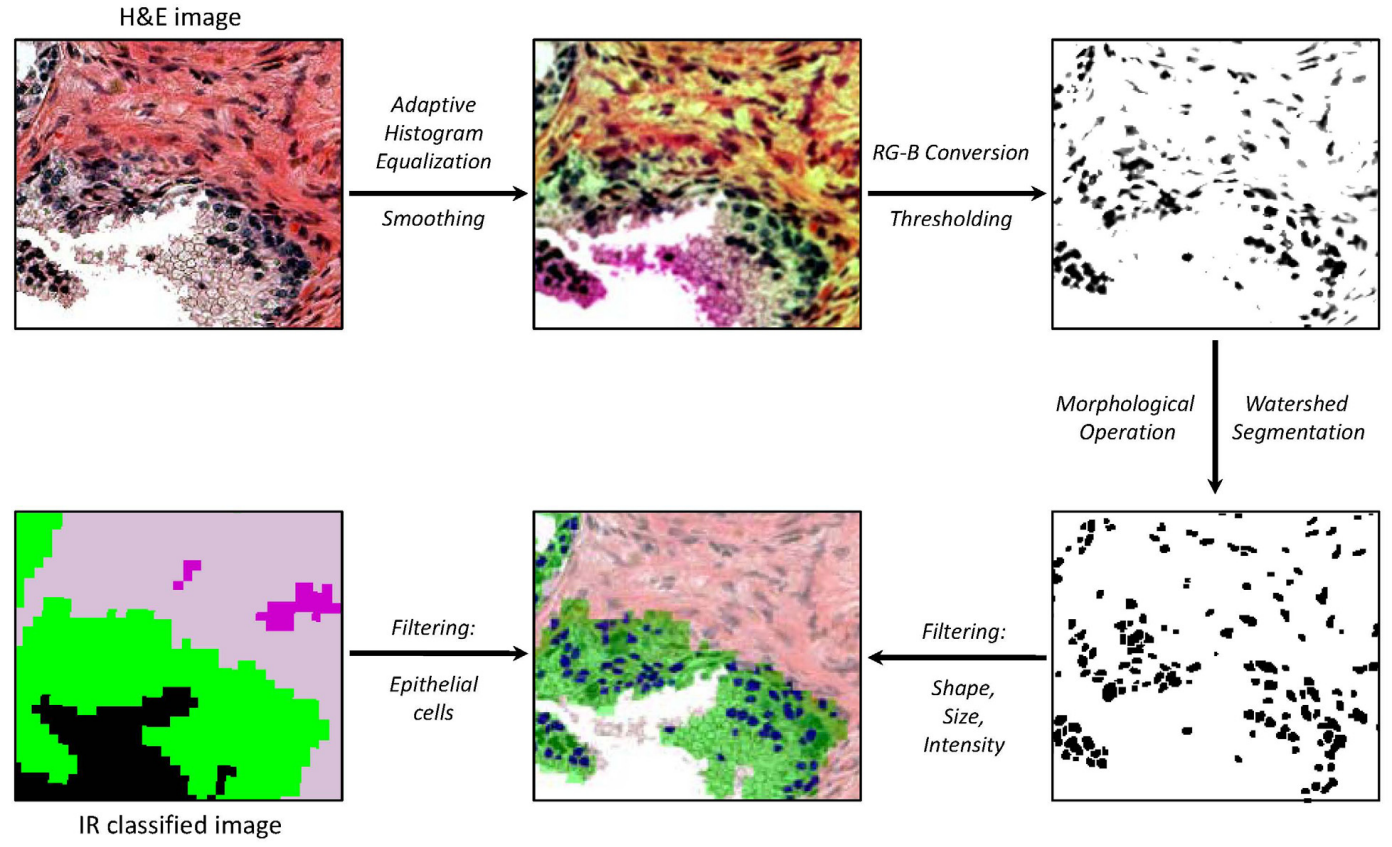
**Nucleus Detection**. Smoothing and adaptive histogram equalization are performed to alleviate variability in H&E stained image and to obtain better contrast. "RG - B" conversion followed by thresholding characterizes the areas where nuclei exist. Morphological closing operation is performed to fill holes and gaps within nuclei, and a watershed algorithm segments each individual nuclei. The segmented nuclei are constrained by their shape, size, and average intensity and epithelial cell classification (green pixels) provided by the overlaid IR image.

Epithelial nucleus detection by automated analysis is more difficult than lumen detection due to variability in staining and experimental conditions under which the entire set of H&E images were acquired. Differences between normal and cancerous tissues, and among different grades of cancerous tissues, also hamper facile detection. To handle such variations and make the contrast of the images consistent, we perform smoothing [43] and adaptive histogram equalization [44] prior to nuclei identification. Nuclei are relatively dark and can be modeled as small elliptical areas in the stained images. This geometrical model is often confounded as multiple nuclei can be so close as to appear like one large, arbitrary-shaped nucleus. Also, small folds or edge staining around lumens can make the darker shaded regions difficult to analyze. Here, we exploit the information provided by the IR classified image to limit ourselves to epithelial cells, and use a thresholding heuristic on a color space-transformed image to identify nuclei with high accuracy. Superimposing the IR classified image on the H&E image, pixels corresponding to epithelium can be identified on the H&E image. These epithelial pixels are dominated by one of two colors: blue or pink, which arise from the nuclear and cytoplasmic component respectively. For nuclei restricted to epithelial cells in this manner, a set of general observations were made that led us to convert the stained image to a new image where each pixel has an intensity value |R + G - B|. (R, G, and B represent the intensity of Red, Green, and Blue channels, respectively.) This transformation, followed by suitable thresholding, was able to successfully characterize the areas where nuclei are present. The threshold values are adaptively determined for Red and Green channels due to the variations in the color intensity. Finally, filling holes and gaps within nuclei by a morphological closing operation [45], the segmentation of each nucleus is accomplished by using a watershed algorithm [45] followed by elimination of false detections. The size, shape, and average intensity are considered to identify and remove artifactual nuclei. Figure 5 details the nucleus detection procedure. (See [Additional file 1: Nucleus Detection] for details.)

### Feature Extraction

**Figure 6:**
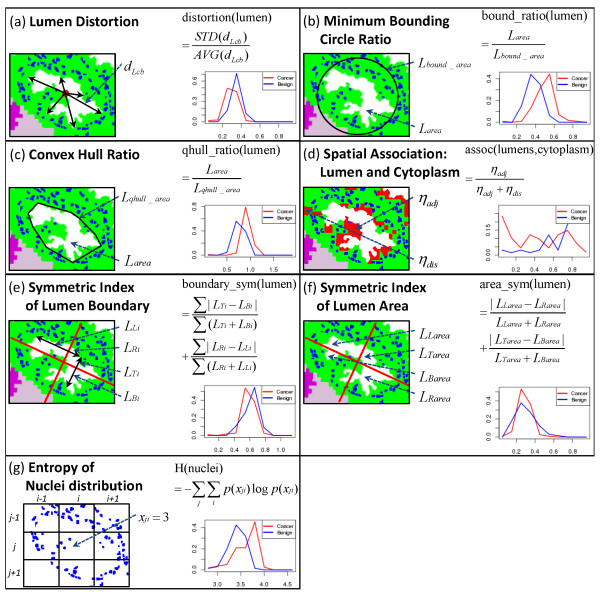
**Examples Features**. Each panel shows one example feature, along with the distributions of the feature's values for cancer (red) and benign (blue) classes.

As mentioned above, the characteristics of nuclei and lumens change in cancerous tissues. In a normal tissue, epithelial cells are located mostly in thin layers around lumens. In cancerous tissue, these cells generally grow to fill lumens, resulting in a decrease in the size of lumens, with the shape of lumens becoming more elliptical or circular. The epithelial association with a lumen becomes inconsistent and epithelial foci may adjoin lumens or may also exist without an apparent lumen. Epithelial cells invading the extra-cellular matrix also result in a deviation from the well-formed lumen structure; this is well-recognized as a hallmark of cancer. Due to filling lumen space and invasion into the extra-cellular space, the number density of epithelial cells increases in tissue. The size of individual epithelial cells and their nuclei also tend to increase as malignancy of a tumor increases. Motivated by such recognized morphological differences between normal and cancerous tissues, we chose to use epithelial nuclei and lumens as the basis of the several quantitative features that our classification system works with. (See examples of such features in Figure 6.) It is notable that these observations are qualitative in actual clinical practice and have not been previously quantified.

#### Epithelial cell-related features

Epithelial cell information is available from IR data. However, individual epithelial cells in the tissue are not easily delineated. Therefore, in addition to features directly describing epithelial cells, we also quantify properties of epithelial nuclei, which are available from the segmentation described above. The quantities we measure in defining features are: (1) size of epithelial cells, (2) size of epithelial nuclei, (3) number of nuclei in the tissue sample, (4) distance from a nucleus to the closest lumen, (5) distance from a nucleus to the epithelial cell boundary, (6) number of "isolated" nuclei (nuclei that have no neighboring nucleus within a certain distance), (7) number of nuclei located "far" from lumens, and (8) entropy of spatial distribution of nuclei (Figure 6G). [Additional file 1: Epithelium-related Features] provide specifics of these measures and their calculation.

#### Lumen-related features

Features describing glands have been shown to be effective in PCa classification [21,25]. Here, we try to characterize lumens and mostly focus on the differences in the shape of the lumens. The quantities we measure in defining these features are: (1) size of a lumen, (2) number of lumens, (3) lumen "roundness" [25], defined as $\frac{L_{peri}}{2L_{area}}r$ where *L_peri _*is the perimeter of the lumen, *L_area _*is the size of the lumen (i.e., number of pixels in the lumen), and *r *is the radius of a circle of size *L_area_*, (4) lumen "distortion" (Figure 6A), computed as $\frac{STD(d_{L_{cb}})}{AVG(d_{L_{cb}})}$ where $d_{L_{cb}}$ is the distance from the center of a lumen to the boundary of the lumen and *AVG(·) *and *STD(·) *represent the average and standard deviation, (5) lumen "minimum bounding circle ratio" (Figure 6B), defined as the ratio of the size of a minimum bounding circle of a lumen to the size of the lumen, (6) lumen "convex hull ratio" (Figure 6C), which is the ratio of the size of a convex hull of a lumen to the size of the lumen, (7) symmetric index of lumen boundary (Figure 6E, see [Additional file 1: Lumen-related Features]), (8) symmetric index of lumen area (Figure 6F, see [Additional file 1: Lumen-related Features]), and (9) spatial association of lumens and cytoplasm-rich regions (Figure 6D, see [Additional file 1: Lumen-related Features]). Features (3) - (8) are various ways to summarize lumen shapes, while feature (9) is motivated by the loss of functional polarization of epithelial cells in cancerous tissues.

#### Global & local tissue features

**Figure 7:**
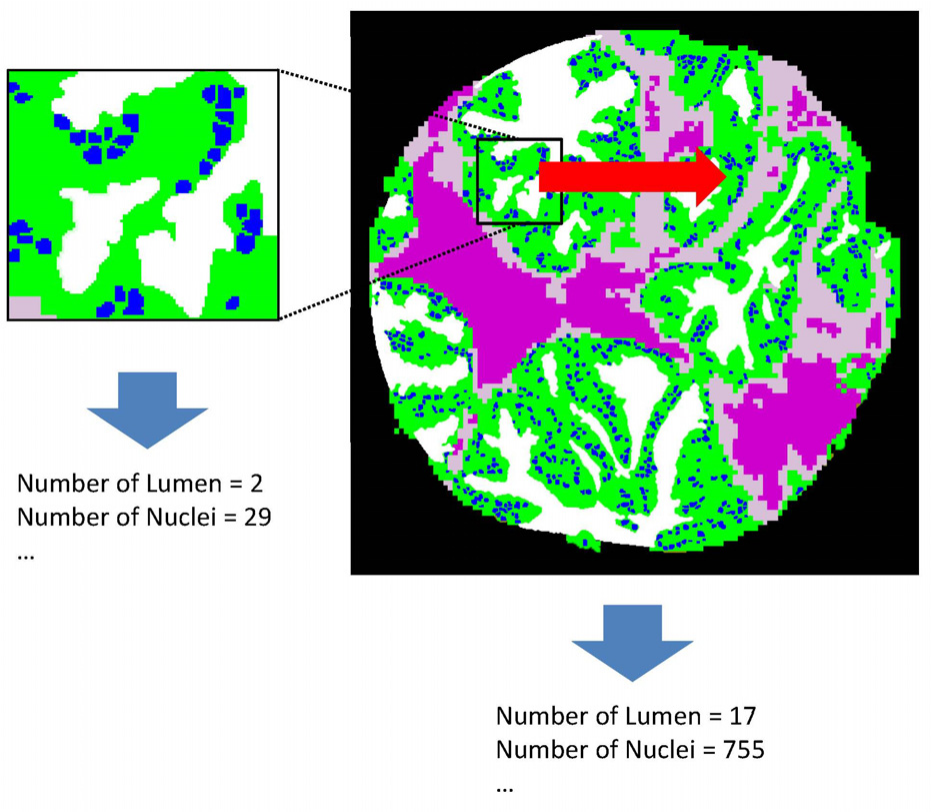
**Global and Local Feature Extraction**. Global features are extracted from the entire tissue sample, and local features are extracted by sliding a window of a fixed size across the tissue sample and computing summary statistics, such as standard deviation, of window-specific scores. In this example, the global feature "number of nuclei" has value 755, while one example position of the sliding window is shown, with "number of nuclei" = 29.

We have described above the individual measures of epithelium and lumen related quantities that form the basis of the features used by our classification system. Normally, these features have to be summary measures over the entire tissue sample or desired classification area. Hence, we employ average (AVG) or standard deviation (STD), and in some cases the sum total (TOT) of these quantities for further analysis. These features are called "global" features since they are calculated from the entire tissue sample. However, in some cases global features may be misleading, especially where only a part of the tissue sample is indicative of cancer. Therefore, in addition to global features, we define "local" features by sliding a rectangular window of a fixed size (100 × 100 pixels) throughout a tissue sample. For each window, AVG and/or TOT of the epithelium and lumen related quantities are computed. STD or extremal values (MIN or MAX) of the AVG and/or TOT values over all windows become local feature values (Figure 7). In all, 67 features (29 global and 38 local features) are defined capturing various aspects of tissue morphology.

### Feature Selection

Feature selection is the step where the classifier examines all available features (67 in our case) with respect to the training data, and selects a subset to use on test data. This selection is generally based on the criterion of high accuracy on training data, but also strives to ensure generalizability beyond the training data. We adopt a two-stage feature selection approach here. In the first stage, we generate a set of candidate features (*C_candidate_*) by using the so-called minimum-redundancy-maximal-relevance (mRMR) criterion [46] (see [Additional file 1: mRMR]). In each iteration, given a feature set chosen thus far, mRMR chooses the single additional feature that is least redundant with the chosen features, while being highly correlated with the class label. *C_candidate _*is a set of features that is expected to be close to the optimal feature set for a data set and a classifier under consideration. It is constructed as follows. Given a feature set F = (f_1_, ..., f_M_) ordered by mRMR, the area under the ROC curve (AUC) of the set of *i *top-ranked features is computed for varying values of *i*. We limit the value of *i *to be ≤ 30. The feature subset with the best AUC is chosen as the *C_candidate_*. In the second stage, feature selection continues with *C_candidate _*as the starting point, using the sequential floating forward selection (SFFS) method [47]. This method sequentially adds new features followed by conditional deletion(s) of already selected features. Starting with the *C_candidate_*, SFFS searches for a feature *x ∉ C_candidate _*that maximizes the AUC among all feature sets *C_candidate _*∪ {*x*}, and adds it to *C_candidate_*. Then, it finds a feature *x *∈ *C_candidate _*that maximizes the AUC among all feature sets *C_candidate _*- {*x*}. If the removal of *x *improves the highest AUC obtained by *C_candidate_*, *x *is deleted from *C_candidate_*. As long as this removal improves upon the highest AUC obtained so far, the removal step is repeated. SFFS repeats the addition and removal steps until AUC reaches 1.0 or the number of additions and deletions exceeds 20, and the feature set with the highest AUC thus far is chosen as the optimal feature set. The classification capability of a feature set, required for feature selection, is measured by AUC, obtained by cross-validation on the training set. SFFS can be directly applied to the original feature set; however, using mRMR may help to reduce the search space and time and to build the optimal classifier by providing a good initial feature set for SFFS.

### Classification

We note that there are two levels of classification here. In the first, IR spectral data is used to provide histologic images where each pixel has been classified as a cell type. In the second, the measures from H&E images and IR images are used to classify tissue into disease states. For the first classification task, we used a Bayesian classifier built on 18 spectral features. This previously achieved >0.99 AUC on cell type classification [48,49]. For the latter task, we used a well established classification algorithm, namely support vector machine (SVM) [50]. As a kernel function, a radial basis function $K(x_{i},x_{j})=\exp\left(-\gamma {\left\|x_{i}-x_{j}\right\|}^{2}\right)$ with parameter γ = 10, 1, 0.1, 0.01, 0.001 is used. Two cost factors are introduced to deal with an imbalance in training data [51]. The ratio between two cost functions was chosen as

$$\frac{C_{+}}{C_{-}}=\frac{\text{number of negative training examples}}{\text{number of positive training examples}}$$

to make the potential total cost of the false positives and the false negatives the same. (See [Additional file 1: SVM] for details.)

### Samples and Data preparation

All of the H&E stained images were acquired on a standard optical microscope at 40x magnification. The size of each pixel is 0.9636 um × 0.9636 um. On the other hand, the pixel size of IR images is 6.25 um × 6.25 um.

**Figure 8:**
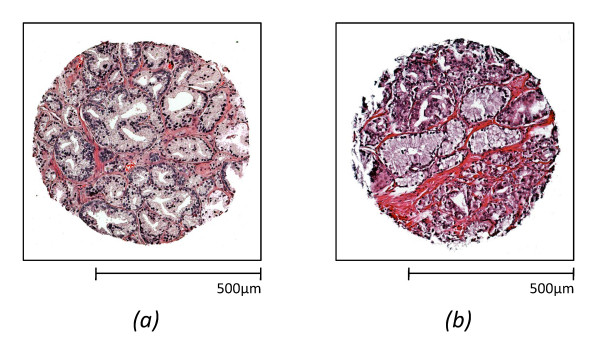
**H&E images of two data sets**. An example of H&E images of (a) *Data1 *and (b) *Data2*. Colors in cytoplasmic and stromal areas are clearly different whereas color of nuclei is less varied.

Two different tissue microarrays were obtained from two different sources (Tissue microarray research program at the National Institutes of Health and Clinomics Inc.). The first data set ("*Data1*") consisted of 240 tissue samples from 180 patients, and the second set ("*Data2*") includes 160 tissue samples from 80 patients. Both sets of tissue samples were sectioned to ~ 7 micron thick sections, with a section being placed on IR transparent BaF2 slides and a serial section on a standard glass slide. The acquisition of data is described elsewhere in [28]. Unfortunately, we were not able to use all of these tissue samples for several reasons. Each data set has two TMAs. One is H&E stained image and the other is IR image. Since these were experimental arrays, some TMA spots were missing in one or both arrays due to processing and plating on the salt plates used for IR analysis. Since our method focuses on epithelial cells, tissue samples which do not have enough epithelial cells (>100) in either of two images (H&E and IR) were not considered in this study. Moreover, some tissue samples in *Data2 *are spatially displaced and fused with neighboring tissue samples. Eliminating those tissue samples, 66 benign tissue samples and 115 cancer tissue samples are remained for *Data1*, and 14 benign and 36 cancer tissue samples remained for *Data2*. An example of H&E images for both data sets is shown in Figure 8.

## Results and discussion

### The classification system achieves AUC greater than 0.97 on both data sets

We first performed *K*-fold cross validation on each data set. The data set was divided into *K *roughly equal-sized partitions, one partition was left out as the "test data", the classifier was trained on the union of the remaining *K - 1 *partitions (the "training data") and evaluated on the test data. This was repeated *K *times, with different choices of the left-out partition. (We set *K *= 10.) In each repetition, cross-validation on the training data was used to select the feature set with the highest AUC as explained in Feature Selection. The correct and incorrect predictions in the test data, across all *K *repetitions, were summarized into a ROC plot and the AUC was computed, along with specificities when sensitivity equals 90, 95, or 99%. Since the cross-validation exercise makes random choices in partitioning the data set, we examined averages of these performance metrics over 10 repeats of the entire cross-validation pipeline. The average AUC for *Data1 *and *Data2 *were 0.982 and 0.974 respectively (Table 1, "feature extraction" = "IR & HE"). At 90%, 95%, and 99% sensitivities, the average specificity achieved on *Data1 *was 94.76%, 90.91%, and 77.80% respectively, while that on *Data2 *was 92.53%, 84.19%, and 49.54% respectively. SVM using the kernel parameter γ = 1 is used here. This result is consistent with the classification results using different values of the parameter γ (See [Additional file 1: Supplementary Table S2] for details). We note that other classification methods can be also used. Among various methods, a logistic model tree [52], which combines linear logistic regression with decision tree induction, was used, and achieved slightly lesser performance than SVM (results not shown here).

**Table 1:** Classification results via cross-validation

Data set	Feature Extraction	AUC		Sensitivity (%)	Specificity (%)		**M**_**f**_
		AVG	STD		AVG	STD	
*Data1*	IR & HE	0.982	0.0030	90	94.76	1.64	13
				95	90.91	1.62	
				99	77.80	5.52	
	
	HE only	0.968	0.0052	90	91.64	2.26	11
				95	83.90	1.91	
				99	53.43	13.65	

*Data2*	IR & HE	0.974	0.0145	90	92.53	7.11	7
				95	84.19	10.84	
				99	49.54	22.51	
	
	HE only	0.880	0.0175	90	61.34	10.31	8
				95	22.21	10.06	
				99	11.21	6.01	

AVG and STD denote average and standard deviation across ten repeats of cross-valdiation. M_f _is the median size of the feature set obtained by feature selection from training data. Column "Feature Extraction" indicates if features were obtained using H&E as well as IR data, or with H&E data alone. The parameter γ of a radial basis kernel for SVM is set to 1.

One way to interpret the above values is to examine our automated pipeline as a pre-screening mechanism to identify the samples to be examined by a human pathologist. At a "true positive rate" of 99% (which means that only 1% of the cancer samples will be missed by the screen), the "false positive rate" is 22.2% (i.e., 22.2% of the benign samples will make it through the screen) on average for *Data1 *(Table 1), thereby reducing the workload of the pathologist by 4.5-fold. While the error rate of manual pathology determinations is generally accepted to be in 1-5% range, inclusion of confounding cancer mimickers raises the rate to as high as 7.5% [53]. Also noteworthy is the observation that the same algorithm performs consistently well on both data sets, that were obtained from different staining conditions. This speaks to the robustness of the classification framework, an attribute that we investigated further in the next exercise.

### Classification system is robust to staining conditions

Here, we trained a classifier on *Data1 *and tested its performance on *Data2 *(Table 2, "Data set" = "Test") using SVM with γ = 1. We observed an average AUC of 0.956, with average specificity of 88.57%, 81.92%, and 26.86% at sensitivity equaling 90%, 95%, and 99% respectively (Table 2, "feature extraction" = "IR & HE" and "Data set" = "Test"). These values are competitive with the cross-validation results on *Data2 *(Table 1), where the training and testing were both performed on (disjoint parts of) *Data2*. It should be noted that in Table 2 "Data set" = "Train" means that the classifier was not only trained but also tested on *Data1*, and thus the difference between the "Train" and "Test" rows does not refer to a difference in performance on the two data sets. As a classifier is trained on *Data2 *and tested on *Data1*, we obtained the average AUC of 0.855 and average specificity of 50.18%, 40.41%, and 12.33% at sensitivity equalling 90%, 95%, and 99% respectively ([Additional file 1: Supplementary Table S4, γ = 1]). The results are worse than both the cross-validation results on *Data1 *and the validation results on *Data2*. This may be due to the fact that the number of samples in *Data2 *is relatively small and much unbalanced. In addition, varying the parameter value γ of SVM, the results, by and large, are the same (See [Additional file 1: Supplementary Table S3 and S4] for details).

**Table 2:** Validation between data sets

Feature Extraction	Data set	AUC		Sensitivity (%)	Specificity (%)		**M**_**f**_
		AVG	STD		AVG	STD	
IR & HE	Train	0.994	0.0006	90	98.30	0.68	13
				95	96.58	1.10	
				99	91.55	2.55	
		
	Test	0.956	0.0089	90	88.57	5.96	
				95	81.92	5.28	
				99	26.86	15.50	

HE only	Train	0.986	0.0021	90	97.77	0.97	10
				95	91.56	2.49	
				99	79.29	4.47	
		
	Test	0.918	0.0100	90	65.51	8.37	
				95	46.14	7.53	
				99	13.29	6.94	

A classifier is trained on *Data1 *and tested on *Data2*. AVG and STD denote the average and standard deviation. M_f _is the median size of the optimal feature set. Column "Feature Extraction" indicates if features were obtained using H&E as well as IR data, or with H&E data alone. Column "Data set" indicates if the performance metrics are from training data (*Data1*) or from test data (*Data2*). The parameter γ of a radial basis kernel for SVM is set to 1.

### Use of IR data improves classification performance

To assess the utility of the IR-based cell-type classification, we repeated the above exercises after extracting features without the guidance of the IR data; i.e., epithelial cells were predicted from the H&E images alone (see [Additional file 1: Epithelium Detection] for details). All of the features defined in Feature Extraction were used, except for "Spatial association of lumens and apical regions", since the distinction between cytoplasm-rich and nuclear-rich region in epithelial cells was unclear in H&E images. The results from this disadvantaged classifier are shown in Tables 1 and 2 ("feature extraction" = "HE only"). For both types of experiments, we obtained lower average AUCs and specificity values. For instance, the AUC of cross-validation in *Data2 *(Table 1) dropped from 0.974 to 0.880. Similarly, the results of validation between data sets (Table 2) were substantially worse now compared to the IR-guided classification, with the AUC dropping from 0.956 to 0.918. We also observed that the average AUC dropped in the absence of IR data as using different values of parameter γ for SVM (See [Additional file 1] for details). This indicates that the use of IR data, i.e., the improved epithelial identification, helps to attain better classification performance. We also note that other methods, if any, which could achieve high accuracy identification of epithelial cells may have the same impact with the IR data on the classification.

Previously, Tabesh *et al. *achieved an accuracy of 96.7% via cross validation in cancer/no-cancer classification [22]. Color, morphometric, and texture features were extracted, and all images were acquired under similar conditions. We note that our classification result (Table 1), based solely on morphology, is comparable to their result; however the software developed by Tabesh et al. was not available for evaluation in our data sets. Color and texture features could provide additional information; however, their robustness to different data sets is questionable, and their interpretation is not as obvious as that of morphological features, which are used in clinical practice. Different data sets may have varied properties which may be attributable to staining variations, inconsistent image acquisition settings, and image preparation. The performance of the same method based on texture features has been seen to greatly change from one data set to another [19,22,25]. Variations in staining may affect color features. In contrast, morphological features were shown to be robust to varying image acquisition settings [25]. Nonetheless, the quality of morphological features is subject to segmentation of histologic objects. Thus, any method based on morphological features will benefit from the IR cell-type classification.

### Examination of discriminative features

**Figure 9:**
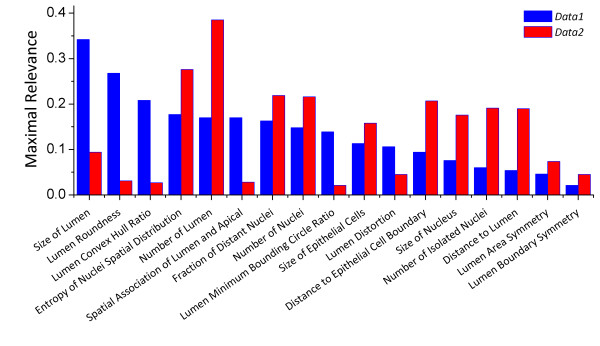
**Importance of 17 feature categories**. The average "maximal relevance" of features belonging to each feature category is shown, for both data sets, sorted in decreasing order for the first data set.

**Figure 10:**
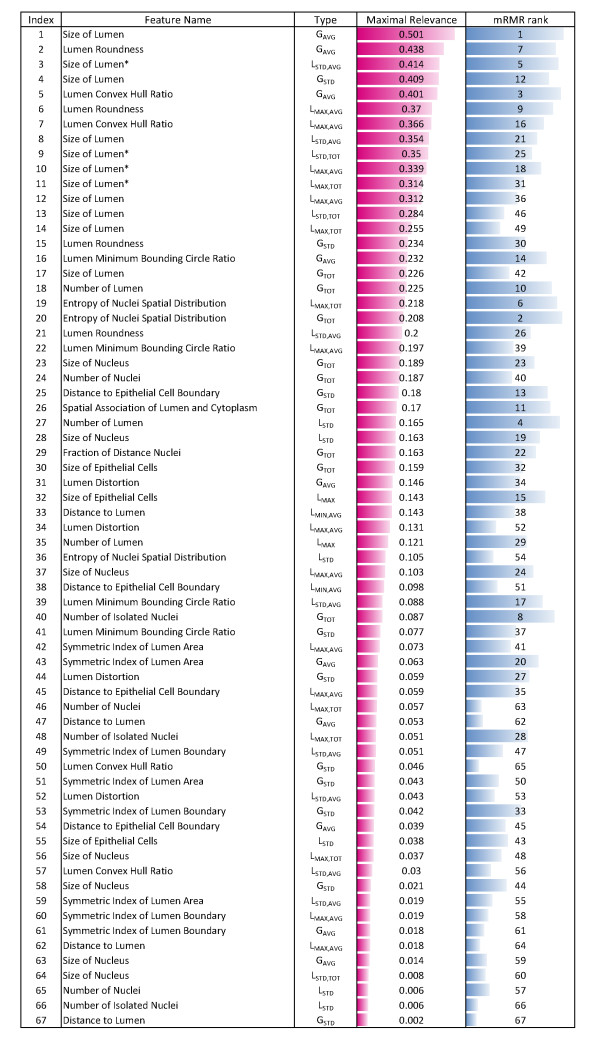
**List of features and their maximal relevance and "mRMR rank"**. In the second column, *G *and *L *represent global and local features, respectively. *AVG*, *STD*, *TOT*, and *MAX *denote the average, standard deviation, total amount, and extremal value of features. * In computing local features representing "size of lumen", two options are available: one is to consider only the part of the lumen within the window, and the other is to consider the entire lumen into account. Asterisk indicates that the former option was chosen.

We examined the importance of each feature by its rank in the first phase of feature selection, based on its "relevance" to the class label (see [Additional file 1: mRMR]). Since different features (e.g., average or standard deviation, global or local features) based on the same underlying quantity (e.g., "lumen roundness") generally have similar relevance, we examined the average relevance of features in each of 17 feature categories (Figure 9), for each data set. The relevance of features is consistent across cross-validation (see [Additional file 1: Supplementary Figure S1]). The complete list of the individual features and their relevance and mRMR rank (for *Data1*) is available in Figure 10. For *Data1*, lumen-related feature categories are most relevant in general, while epithelium-related feature categories are most important for *Data2*. It is surprising that the top 3 feature categories in *Data1 *(Figure 9, blue bars) - size of lumen, lumen roundness, and lumen convex hull ratio - have very low relevance in *Data2*, although we note that this may be in large part due to variations in staining and malignancy of tumors between the two data sets and differences in the size of two data sets. The comparable classification results on *Data2 *(Table 1, 2), in spite of the maximal relevance differences, may indicate the broadness of our feature set and the accuracy of our feature selection method and facilitate the application of the same classifier on different data sets. Nevertheless, a larger scale study may be necessary to precisely examine the differences between data sets and features. It is, however, noteworthy that examining the features (or feature categories) with highest relevance alone may be slightly misleading, because this examination does not account for redundancy among features.

**Figure 11:**
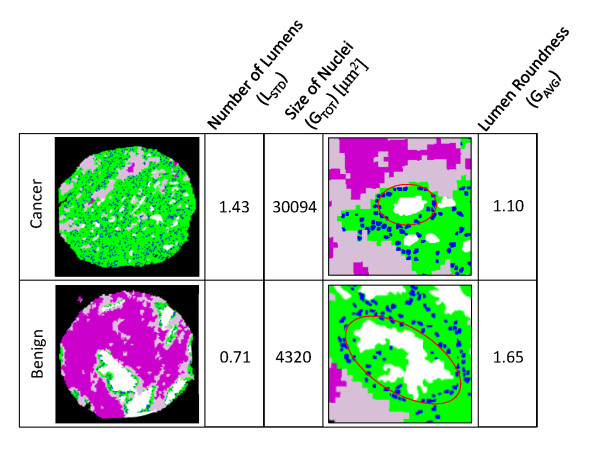
**Optimal features for distinguishing cancer and benign tissue samples**. The three features shown here are most frequently present in the optimal feature set chosen by the classifier.

To further examine the most informative and non-redundant features, we inspected the optimal feature sets selected after both stages of the feature selection component. For both *Data1 *and *Data2*, the selection of the features is consistent across all folds of cross validation. (See [Additional file 1: Supplementary Figure S2] for details.) In Figure 11, we show an example of three most frequently selected features for *Data1*: number of lumens (L_STD_), lumen roundness (G_AVG_), and size of nucleus (G_TOT_). We note that these include both lumen and epithelium related features. Lumen roundness (G_AVG_) is the only one ranked high by maximal relevance (Figure 10), yet all three features are consistently chosen by the classifier, since they provide different, complementary information on a tissue: greater circularity of lumens and increase in the number of lumens and the size of nuclei indicate malignancy of a tissue.

## Conclusions

In this manuscript, we have presented a means to eliminate epithelium recognition deficiencies in classifying H&E images for presence or absence of cancer. The method is entirely transparent to a user and does not involve any adjustment or decision-making based on spectral data. We were able to achieve very effective fusion of the information from two different modalities, namely optical and IR microscopy, that provide very different types of data with different characteristics. Several features of the tissue were quantified and employed for classification. We found that robust classification could be achieved using a few measures, which are detailed to arise from epithelial/lumen organization and provide a reasonable explanation for the accuracy of the model. The choice of combining the IR and optical data is shown to be necessary for achieving the high accuracy values observed. We anticipate that the combined use of the two microscopies - structural and chemical - will lead to an accurate, robust and automated method for determining cancer within biopsy specimens.

## Competing interests

The authors declare that they have no competing interests.

## Authors' contributions

JTK contributed to developing algorithms, programming, and data analysis, and drafted the manuscript. SMH made the diagnoses of tissue samples. SS contributed to developing algorithms and data analysis. RB contributed to the data preparation and data analysis. All authors revised the manuscript and approved the final version.

## Pre-publication history

The pre-publication history for this paper can be accessed here:

http://www.biomedcentral.com/1471-2407/11/62/prepub

## Supplementary Material

Additional file 1 **Supplementary material**. It includes detailed description of image processing, feature extraction, feature selection, and classification method and results. Click here for file
