# Supplementary material for: Multimodal microscopy for automated histologic analysis of prostate cancer
Source: BMC Cancer. 2011 Feb 9;11:62. doi: 10.1186/1471-2407-11-62 (PMC3045985; doi:10.1186/1471-2407-11-62)
Supplement: Additional file 1 — Supplementary material. It includes detailed description of image processing, feature extraction, feature selection, and classification method and results. [file 1471-2407-11-62-S1.DOCX]

**Image Registration**

In order to map the cell type information from IR classified images on H&E images, the image registration, the process of finding the optimal spatial and intensity transformation [1] of one image (H&E image; *I_reference_*) to the other (IR classified image; *I_target_*), is indispensable. Two tissue samples were physically in the same intact tissue and are structurally similar. Macroscopic sample shape and empty space (lumens) inside the samples are well matched between two images. However, due to differences in two imaging techniques, two images have different properties (total image and pixel size, contrast mechanisms and data values) and specify different information; H&E images provide detailed morphological information whereas IR classified images contain cell type information. Intensity values of each pixel in two images are also greatly different. Each pixel in H&E images has 3 channels (Red, Green, and Blue) ranging from 0 to 255, and, in IR classified images, a label indicating its cell type is assigned to each pixel.

From above observations, we decided to eliminate intensity differences prior to registration, to use an affine transformation [1] as a spatial transformation, and to estimate the transformation from the entire image. To eliminate the intensity difference between H&E image and IR classified image, we convert both images into binary images, i.e., pixels representing a tissue are assigned “1” and other pixels including lumens are set to “0”. For IR classified image, pixels labeled with cell types is the ones representing a tissue. Accordingly, assigning “1” to those pixels and “0” to others completes the binarization. For H&E image, we use a proper threshold value (> 200) for the intensity of Red (R), Green (G), and Blue (B) channels since both lumens and background regions are white. Then, inverting the thresholded image gives the binary image of H&E image. As a result of the binarization, the intensity transformation is unnecessary. It should be noted that the binarization does not alter the geometrical characteristics (macroscopic shape and lumens) of the two images. The affine transformation (*f*) transforms a coordinate (*x_1_*, *y_1_*) to the (*x_2_*, *y_2_*) coordinate after translations (*t_x_*, *t_y_*), rotation by *θ*, and scaling by factor *s*.

$$\left[\begin{matrix} x_{2} \\ y_{2} \end{matrix}\right]=\left[\begin{matrix} t_{x} \\ t_{y} \end{matrix}\right]+s\left[\begin{matrix} \cos\theta& -\sin\theta\\ \sin\theta& \cos\theta\end{matrix}\right]\left[\begin{matrix} x_{1} \\ y_{1} \end{matrix}\right]$$

Since two adjacent tissue samples are structurally similar, it is assumed that two images do not suffer from large deformation, and the affine transformation is sufficient to model the geometrical change between two images. Difficulty in extracting features, ascribed to different properties and information provided by two images, leads us to use entire image for estimating the transformation parameters. We define absolute intensity difference between two images as error metric (or similarity measure). The absolute intensity difference between two images is, in fact, corresponding to the total number of pixels where two images have different labels owing to binarization. The better registration, the smaller number of those pixels it results in. Thus, the optimal registration can be obtained by minimizing the absolute intensity difference between two images. In other words, the image registration amounts to finding the optimal parameter values of the affine transformation

$$(t_{x}^{*},t_{y}^{*},\theta^{*},s^{*})=\mathop{\mathrm{argmin}}\limits_{t_{x},t_{y},\theta,s} \left|I_{reference}-f\left(I_{target};t_{x},t_{y},\theta,s\right)\right|$$

To reduce the search space, we align the center of two images and scaled up *I_target_* by estimating the radius of both samples. Afterwards, we draw random samples of the parameter values, transform the coordinate of *I_target_*, and compute the absolute intensity difference to obtain the initial solution. Then, the downhill simplex method [2] is applied to attain the final solution.

In order to quantitatively validate the accuracy of the method, we conduct experiments using one IR classified image and simulated images. We generate the simulated images by transforming the given IR classified image with different parameter values: 1) scaling factor *s* in the range [0.5, 1.5], 2) rotation angle *θ* in the range [$-\frac{\pi}{2}$, $\frac{\pi}{2}$], 3) translation (*t_x_*, *t_y_*) in the range [-50, 50]. For each of the three cases, 100 simulated images are generated, and another 100 images are also generated by varying all parameters simultaneously. After applying the registration method to register the IR classified image with the simulated images, we compared the true parameters with the recovered parameters by computing registration error (the absolute difference between parameters). As shown in Table S1, the registration method well recovers the true parameters. Therefore, we expect the registration method to successfully register the H&E image with the IR classified image in the absence of large deformation.

**Lumen Detection**

Complete lumen detection starts from identifying white spots inside the samples from the H&E image by using a proper threshold value (> 200) for the intensity of Red (R), Green (G), and Blue (B) channels. The white spots may include many artifacts which are, in our observations, relatively small and/or have narrowly elongated needle-like shape. Owing to IR overlay, pixels corresponding to epithelial cells from the IR classified image can be mapped on the H&E image, and it allows us to identify artifactual lumens, which are not associated epithelial cells. By definition, lumens are surrounded by epithelial cells. We examine each white spot whether more than 30% of its perimeter is next to or within the areas where epithelial pixels are present. If the condition is not satisfied, the spot is considered to be an artifact. To further prune the white areas that passed the condition, a simple rule, restricting the size and shape, is invoked: If the size of any white area is smaller than 10 pixels or the major and minor axis ratio (r_major/minor_) is greater than 3 when its size is smaller than 100 pixels, the white area also is considered to be an artifact. Lumens are progressively smaller and lesser distorted elliptical or circular with increasing grade; that is, r_major/minor_ is getting closer to 1. Larger r_major/minor_ is indicative of artifact. r_major/minor_ is computed by using the major and minor axes of an ellipse fitted to each white area.

Since each tissue sample is a small portion of an entire tissue, the tissue sample often includes lumens that do not form a complete geometrical shape. We call this kind of lumens incomplete lumens. Their perimeter is adjacent to either epithelial cells or to background. The fraction of the lumen’s perimeter that is adjacent to background is relatively small. However, without examining the original tissue that the samples were taken from, it is impossible to infer the original size and shape of such incomplete lumens. To handle this problem, we model an entire tissue sample as a circle, and the white spots between the tissue sample and the circle are the candidate incomplete lumens. The same threshold value (> 200) for the complete lumen detection is used to identify candidate white areas which may include artifactual lumens. The artifactual incomplete lumens are relatively small and/or in crescent shapes along the edge of tissues. Crescent-like artifacts result from the gaps between the tissue sample and the circle fitted to the sample, and their average distance from the center of the sample is close to the radius of the sample. Based on these observations, similar to the artifactual complete lumens, we restrict the white areas by the following considerations: the fraction of their perimeter bordering epithelial cells must be > 0.65 and that bordering background must be < 0.4, their size must be greater than 100 pixels, the shape must have r_major/minor_ < 3, and the average distance of their perimeter to the center of the tissue must be less than 90% the radius of the tissue core.

**Nucleus Detection**

Owing to variability in staining, experimental conditions, and status of tissues, more cautious and meticulous techniques are required to detect nuclei. Here, nuclei are modeled as relatively dark and small elliptical areas in the stained images. In our observations, both blue and red channel intensity of pixels corresponding to epithelial cells, nuclear components in particular, do not suffer from the variability as much as green channel intensity. The green channel intensity varies a lot from image to image; for example, its histogram is highly skewed in cancerous tissues. This may increase a false discovery of nuclei in cancerous cells. To overcome the problem, make the segmentation consistent and robust, and obtain better contrast, we smooth the stained image [3] and apply adaptive histogram equalization [4] to green channel. Adaptive histogram equalization is an image enhancement technique which redistributes each pixel value proportional to the intensities of its surrounding pixels. Because applying adaptive histogram equalization to all the three channels could bring dramatic alterations and biases in color spaces, we opt to apply to only green channel possessing the highest deviation. As mentioned above, epithelial pixels mapped on the stained images using the IR overlay can provide nuclear and cytoplasmic pixels. Examining nuclei restricted to epithelial cells, a set of general observations may be noted: 1) Red, Green, and Blue channel intensities are lower in nuclear pixels and higher in cytoplasmic pixels. 2) Green channel intensity is lower than other channels in both cytoplasmic and nuclear pixels. 3) In stromal cells, which are not considered here, Red channel intensity is usually higher than other channels. 4) A difference between Red and Blue channel intensities is small both in cytoplasmic and nuclear pixels. Based on these observations, we found that converting the stained image to a new image where each pixel has an intensity value |R + G – B| could well characterize the areas where epithelial nuclei are present; nuclear pixels mostly have lower values than cytoplasmic pixels and pixels belonging to other cell types such as stroma. During the color transformation, a few intensity constraints are imposed on Green and Red channels. For both Green and Red channels, the threshold values (*Th_Red_* and *Th_Green_*) are computed by $AVG(P)-\frac{2}{3}STD(P)$, respectively. *P* represents a set of pixels where Red channel intensity is less than either of two other channels (avoid to include stromal pixels) and *AVG(·)* and *STD(·)* represent the average and standard deviation. Adaptively computed threshold values may help to manage variations in the stained images. Green channel intensity is required to be less than *Th_Green_* and Red channel intensity is required to be less than *Th_Red_* or other two channel intensities. Restriction imposed on Red channel is to eliminate pixels corresponding to stromal cells, just in case that the IR overlay fails. After the color conversion, we apply a morphological closing operator [5] to the image to fill small holes and gaps within nuclei, and the segmentation of each individual nucleus is accomplished by using watershed algorithm [5]. To alleviate possible over-segmentation of the nuclei [6], we expand each segmented nucleus area *N_seg_* by including all neighboring pixels whose intensities falling within $AVG(N_{seg})\pm STD(N_{seg})$. Although properly determined, the segmentation may include many false predictions. To refine the segmentation, each individual nucleus is constrained by its shape and size: r_major/minor_ < 4 and size of a nuclus > 5 and < 2 $\times$ median size of all nuclei. In addition, the average intensity of a nucleus is restricted to be less than *Th_Green_*. The nuclei that satisfy all the conditions and are located within the epithelial cells are reported as epithelial nuclei.

**Epithelium Detection**

In epithelial cells, two types of pixels can be observed – nuclear and cytoplasmic pixels. Our strategy to detect epithelial pixels from the H&E stained images is essentially to identify cytoplasmic pixels since nuclei can be detected by the above method. The set of observations made for epithelial cells above is useful for cytoplasmic pixel detection. In addition to the observations, it is noted that the ratio of blue channel intensity to sum of all channel intensity is quite high for cytoplasmic pixels. Hence, we compute the value of each pixel as follows:

$$B\left(1+\frac{B}{R+G+B}\right)-G$$

It emphasizes the pixels that have both higher intensity and relatively higher ratio of blue channel and have lower green channel intensity, and such pixels are cytoplasmic pixels in general. The segmentation of cytoplasmic areas is performed by finding a threshold value iteratively [7]. At iteration *i*, a threshold value is updated as $T_{i}=\frac{1}{2}\left(\mu_{i}^{1}+\mu_{i}^{2}\right)$where *μ_i_^1^* and *μ_i_^2^* denote the average values of two sets of pixels grouped by the threshold value *T_i-1_*. One set contains the pixels whose values are greater than *T_i-1_* (cytoplasmic areas) and the pixels in the other set has the values less than *T_i-1_*. The thresholding method may not capture all the cytoplasmic areas. We grow each cytoplasmic area *C_seg_* by finding the adjacent pixels within $AVG(C_{seg})\pm\frac{1}{2}STD(C_{seg})$. We further indentify and fill small holes inside of each segment to include pixels representing epithelial nuclei. The segmented image often contains many salt and pepper type noise. To remove them, median filter [8] is applied to the segmented image. As did in nucleus detection, adaptive histogram equalization is applied to green channel to deal with variability in the stained images prior to epithelium detection.

**Epithelium-related Features**

List of names and meanings of epithelium related features are:

1) Size of Epithelial cells: Size of epithelial cells.

2) Size of a Nucleus: Size of a nucleus.

3) Number of Nuclei: Number of nuclei.

4) Distance to Lumen: Distance from the center of a nucleus to the boundary of the closest lumen.

5) Distance to Epithelial Cell Boundary: Epithelial cell boundaries are estimated by drawing a Voronoi diagram of the segmented epithelial regions (obtained from IR image) with the segmented nuclei serving as the Voronoi sites. The cell corresponding to each nucleus, also called the Voronoi cell, comprises all points that are closer to that nucleus than to any other nuclei. The Voronoi cell of a nucleus is considered as the epithelial cell to which the nucleus belongs, and the distance to the epithelial cell boundary is the distance from the center of the nucleus to the boundary of its Voronoi cell.

6) Number of Isolated Nuclei [9]: Number of nuclei without having a neighboring nucleus within a distance D_Iso_ (20 um) from the center of each nucleus.

7) Fraction of Distant Nuclei: Fraction of nuclei away from lumens. If the distance from a nucleus to the boundary of the closest lumen is greater than D_Dis_ (30 um), the nucleus is called a distant nucleus.

8) Entropy of Nuclei Spatial Distribution: To measure the entropy of nuclei spatial distribution, an entire tissue is divided into N $\times$ N equal-sized partitions and the number of nuclei in each partition is counted. The entropy is computed as follows:

$$H(Nuclei)=-\sum_{i=1}^{n} \sum_{j=1}^{n} p(x_{ij})\log p(x_{ij})$$

p() denotes the probability mass function of the number of nuclei in a partition. *x_ij_* denotes the number of nuclei in (i,j)th partition.

**Lumen-related Features**

List of names and meanings of lumen related features are:

1) Size of a Lumen: Number of pixels in a lumen.

2) Number of Lumens: Number of lumens in a tissue.

3) Lumen Roundness [10]: Roundness of a lumen is defined as $\frac{L_{peri}}{2L_{area}}r$ where $L_{peri}$ is the perimeter of the lumen, $L_{area}$ is the size of the lumen, and $r$ is the radius of a circle with the size of $L_{area}$.

4) Lumen Distortion: Distortion of a lumen is computed as $\frac{STD(d_{Lcb})}{AVG(d_{Lcb})}$ where $d_{Lcb}$ is the distance from the center of a lumen to the boundary of the lumen.

5) Lumen Minimum Bounding Circle Ratio: Ratio of the size of a minimum bounding circle of a lumen to the size of the lumen.

6) Lumen Convex Hull Ratio: Ratio of the size of a convex hull of a lumen to the size of the lumen.

7) Symmetric Index of Lumen Boundary: Sum of Vertical and Horizontal Symmetry. Vertical and Horizontal Symmetry are defined as $\frac{\sum\left|L_{Ti}-L_{Bi}\right|}{\sum\left(L_{Ti}+L_{Bi}\right)}$ and $\frac{\sum\left|L_{Ri}-L_{Li}\right|}{\sum\left(L_{Ri}+L_{Li}\right)}$, respectively. $L_{Ti}$ and $L_{Bi}$ are vertical distances from a vertical axis to the boundary of the lumen, $L_{Li}$ and $L_{Ri}$ are horizontal distances from a horizontal axis to the boundary of the lumen. The vertical axis runs along the longest diameter, and the horizontal axis runs perpendicularly to the horizontal axis passing the center of the lumen.

8) Symmetric Index of Lumen Area: Sum of Left-Right Area Symmetry and Top-Bottom Area Symmetry. Left-Right and Top-Bottom Area symmetry are computed as $\frac{\left|L_{Larea}-L_{Rarea}\right|}{L_{Larea}+L_{Rarea}}$ and $\frac{\left|L_{Tarea}-L_{Barea}\right|}{L_{Tarea}+L_{Barea}}$, respectively. $L_{Larea}$, $L_{Rarea}$, $L_{Tarea}$, and $L_{Barea}$ are the size of left, right, top, and bottom quadrants, respectively. These quadrants are obtained by dividing the lumen through imaginary vertical or horizontal axes. The vertical and horizontal axes are defined as in 7).

9) Spatial Association of Lumens and cytoplasm-rich regions: Spatial association of lumens and cytoplasm-rich regions is computed as $\frac{\eta_{adj}}{\eta_{adj}+\eta_{dis}}$ where $\eta_{dis}$ is a set of cytoplasm-rich pixels distant to lumens and $\eta_{adj}$ is a set of cytoplasm-rich pixels adjacent to lumens. The process of obtaining cytoplasm-rich pixels is provided in [11]. To obtain adjacent cytoplasm-rich pixels, we first searched the pixels around the boundary of lumens, and if a cytoplasm-rich pixel is found, then neighboring cytoplasm-rich pixels are searched. These are repeated until no more cytoplasm-rich pixels are found.

**mRMR**

mRMR [12] is a feature selection method based on mutual information. It attempts not only to maximize the relevance between selected features and a class label, but also to minimize the redundancy between selected features. Since a set of best features does not result in the best feature set, eliminating redundant features is important to provide a good subset of features. Both relevance and redundancy are characterized in terms of mutual information as follows:

*maximal relevance*: $\max D(S,c),\text{ }D=\frac{1}{\left|S\right|}\sum_{x_{i}\in S} I(x_{i};c)$

*minimal redundancy*: $\max R(S),\text{ }R=\frac{1}{\left|S\right|^{2}}\sum_{x_{i},x_{j}\in S} I(x_{i};x_{j})$

where *I(x,y)* is the mutual information of two variables *x* and *y*, *S* is the feature set, and *c* is the class label. To achieve the goal of optimizing above two conditions simultaneously, the simple mRMR criterion, $\max\left(D-R\right)$, is invoked. It starts from a feature with the highest maximal relevance, and a new feature is selected and added to the current feature set if it satisfies the mRMR critetion among the rest of features. Thus, it generates, in fact, the order of the features according to the mRMR criterion.

**SVM**

Given input data with two classes (+1, –1), SVM [13] constructs a separating hyperplane which aims at maximizing the margin between two classes. Constructing the hyperplane is equivalent to minimizing the *structural risk* function given by

$$V(\omega,b,\xi)=\frac{1}{2}\omega^{T}\omega+C\sum_{i=1}^{n} \xi_{i}$$

$$\text{subject to: }y_{i}\left[\omega x_{i}+b\right]\geq1-\xi_{i}\text{ and }\xi_{i}>0,\text{ }i=1,...,n$$

where C is a parameter controlling tradeoff between training error and model complexity, $y_{i}$ is a class label, $\xi_{i}$ is slack variable, and *n* is the number of training examples. It is known that the dual representation of the above problem is easier to solve and given by

$$\text{minimize: }W(\alpha)=-\sum_{i=1}^{n} \alpha_{i}+\frac{1}{2}\sum_{i=1}^{n} \sum_{j=1}^{n} y_{i}y_{j}\alpha_{i}\alpha_{j}\left(x_{i}\cdot x_{j}\right)$$

$$\text{subject to: }\sum_{i=1}^{n} y_{i}\alpha_{i}\text{ and }0\leq\alpha_{i}\leq C,\text{ }i=1,...,n$$

where $\alpha_{i}$ is a Lagrange multiplier. SVM was originally proposed as a linear classifier, but it could learn a non-linear classifier by replacing the inner-products $\left(x_{i}\cdot x_{i}\right)$ by a kernel function $K\left(x_{i},x_{j}\right)$. In this study, we use the Radial basis kernel $K\left(x_{i},x_{j}\right)=\exp\left(-\gamma\left\|x_{i}-x_{j}\right\|^{2}\right)$ with $\gamma$=10, 1, 0.1, 0.01, 0.001.

An imbalance of positive and negative samples in training data may cause the hyperplane computed by SVM to be biased toward either of two classes. To deal with this problem, different cost factors $C_{+}$ and $C_{-}$ are often introduced in the *structural risk* function to adjust the cost of false positives and false negatives, and the problem becomes [14]

$$\text{minimize: }V(\omega,b,\xi)=\frac{1}{2}\omega^{T}\omega+C_{+}\sum_{i:y_{i}=1}^{n} \xi_{i}+C_{-}\sum_{j:y_{j}=-1}^{n} \xi_{j}$$

$$\text{subject to: }y_{i}\left[\omega x_{i}+b\right]\geq1-\xi_{i}\text{ and }\xi_{i}>0,\text{ }i=1,...,n$$

**Classification Results**

The performance of our method is measured by performing 10-fold cross-validation on each dataset and validation between datasets. Each experiment is repeated by using different values of parameter *γ* for SVM to examine the effect of the value of the parameter. Regardless of the parameter values, we, in general, achieved high classification performance in cross-validation of each dataset (Table S2). >0.96 AUCs were achieved for different values of the parameter except the cross-validation on *Data2* setting *γ*=10 (~0.91 AUC). As a classifier is trained on *Data1* and tested on *Data2*, the classification results were comparable to the cross-validation results on *Data2* over different values of the parameter *γ* (Table S3). Using *γ*=10, an AUC value of ~0.84 was achieved which is slightly worse than others (>0.91 AUC). In the opposite experiments, i.e., a classifier is trained on *Data2* and test on *Data1*, we obtained the AUCs > 0.83 using *γ*=1, 0.1, 0.01, 0.001, and an AUC value of ~0.71 was achieved using *γ*=10. These classification results were substantially worse than the cross-validation results on *Data1* (Table S4). However, this may not indicate the shortcomings of our method, but reveal the poor generalizability of the classifier built on *Data2* due to the small number of samples and its imbalance. For the experiments without the guidance of IR data, the results, by and large, were consistent in varying the parameter *γ*, but significant drop in the AUCs was obtained in comparison with the classification results with the guidance of IR data. In sum, the classification results were not sensitive to the choice of the parameter *γ* except that the AUCs were dropped when *γ*=10.

**Discriminative Features**

At each iteration of cross-validation, the classifier selects the optimal feature set through two-stage feature selection procedure. We examined whether the selected features and their importance are consistent over cross-validation or not. As shown in Figure S1, the maximal relevance of 17 feature categories is consistent within each dataset and over all folds of cross-validation. The features chosen by the classifier are also relatively constant (Figure S2). We note that the median number of the optimal feature set is 13 and 7 for Data1 and Datat2, respectively. Accordingly, more features have higher frequencies in Figure S2 A.

**REFERENCES**

1. Brown LG: **A Survey of Image Registration Techniques.** *Comput Surv* 1992, **24:**325-376.

2. Nelder JA, Mead R: **A Simplex-Method for Function Minimization.** *Comput J* 1965, **7:**308-313.

3. Lee JS: **Speckle Suppression and Analysis for Synthetic Aperture Radar Images.** *Opt Eng* 1986, **25:**636-643.

4. Pizer SM, Amburn EP, Austin JD, Cromartie R, Geselowitz A, Greer T, Terhaarromeny B, Zimmerman JB, Zuiderveld K: **Adaptive Histogram Equalization and Its Variations.** *Comput Vision Graph* 1987, **39:**355-368.

5. Dougherty ER: *An introduction to morphological image processing.* Bellingham, Wash., USA: SPIE Optical Engineering Press; 1992.

6. Roerdink JBTM, Meijster A: **The watershed transform: definitions, algorithms and parallelization strategies.** *Fundam Inf* 2000, **41:**187-228.

7. **Picture Thresholding Using an Iterative Selection Method.** *Systems, Man and Cybernetics, IEEE Transactions on* 1978, **8:**630-632.

8. Huang T, Yang G, Tang G: **A fast two-dimensional median filtering algorithm.** *Acoustics, Speech and Signal Processing, IEEE Transactions on* 1979, **27:**13-18.

9. Tabesh A, Teverovskiy M, Pang HY, Kumar VP, Verbel D, Kotsianti A, Saidi O: **Multifeature prostate cancer diagnosis and Gleason grading of histological images.** *Ieee T Med Imaging* 2007, **26:**1366-1378.

10. Farjam R, Soltanian-Zadeh H, Jafari-Khouzani K, Zoroofi RA: **An image analysis approach for automatic malignancy determination of prostate pathological images.** *Cytom Part B-Clin Cy* 2007, **72B:**227-240.

11. Bhargava R, Fernandez DC, Hewitt SM, Levin IW: **High throughput assessment of cells and tissues: Bayesian classification of spectral metrics from infrared vibrational spectroscopic imaging data.** *Bba-Biomembranes* 2006, **1758:**830-845.

12. Peng HC, Long FH, Ding C: **Feature selection based on mutual information: Criteria of max-dependency, max-relevance, and min-redundancy.** *Ieee T Pattern Anal* 2005, **27:**1226-1238.

13. Vapnik VN: *The nature of statistical learning theory.* New York: Springer; 1995.

14. Morik K, Brockhausen P, Joachims T: **Combining Statistical Learning with a Knowledge-Based Approach - A Case Study in Intensive Care Monitoring.** In *Book Combining Statistical Learning with a Knowledge-Based Approach - A Case Study in Intensive Care Monitoring* (Editor ed.^eds.). pp. 268-277. City: Morgan Kaufmann Publishers Inc.; 1999:268-277.

# Figures


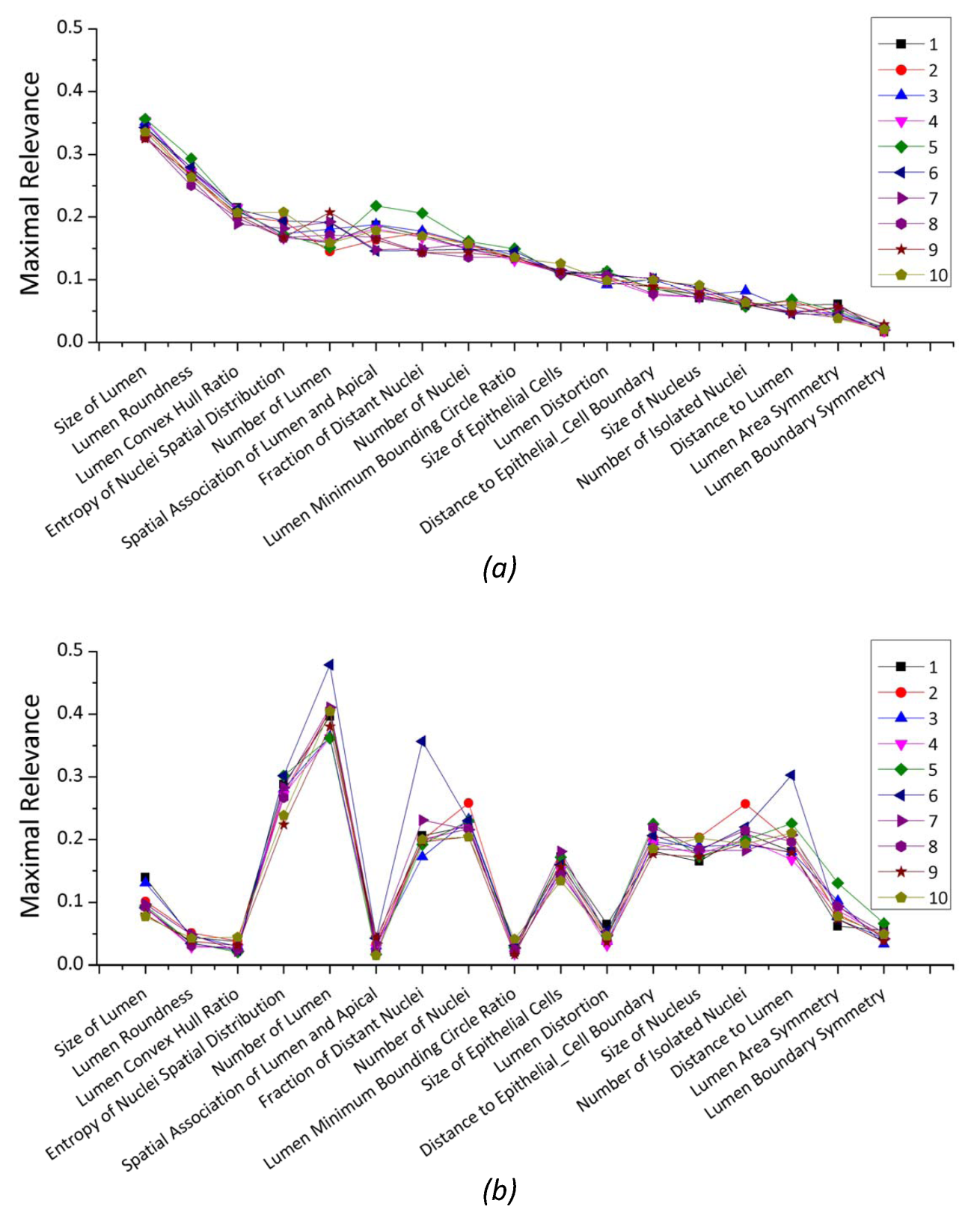


**Figure S1. Importance of 17 feature categories across cross-validation.**

“Maximal relevance” for both datasets (a) *Data1* (b) *Data2* is consistent over all folds of cross-validation.


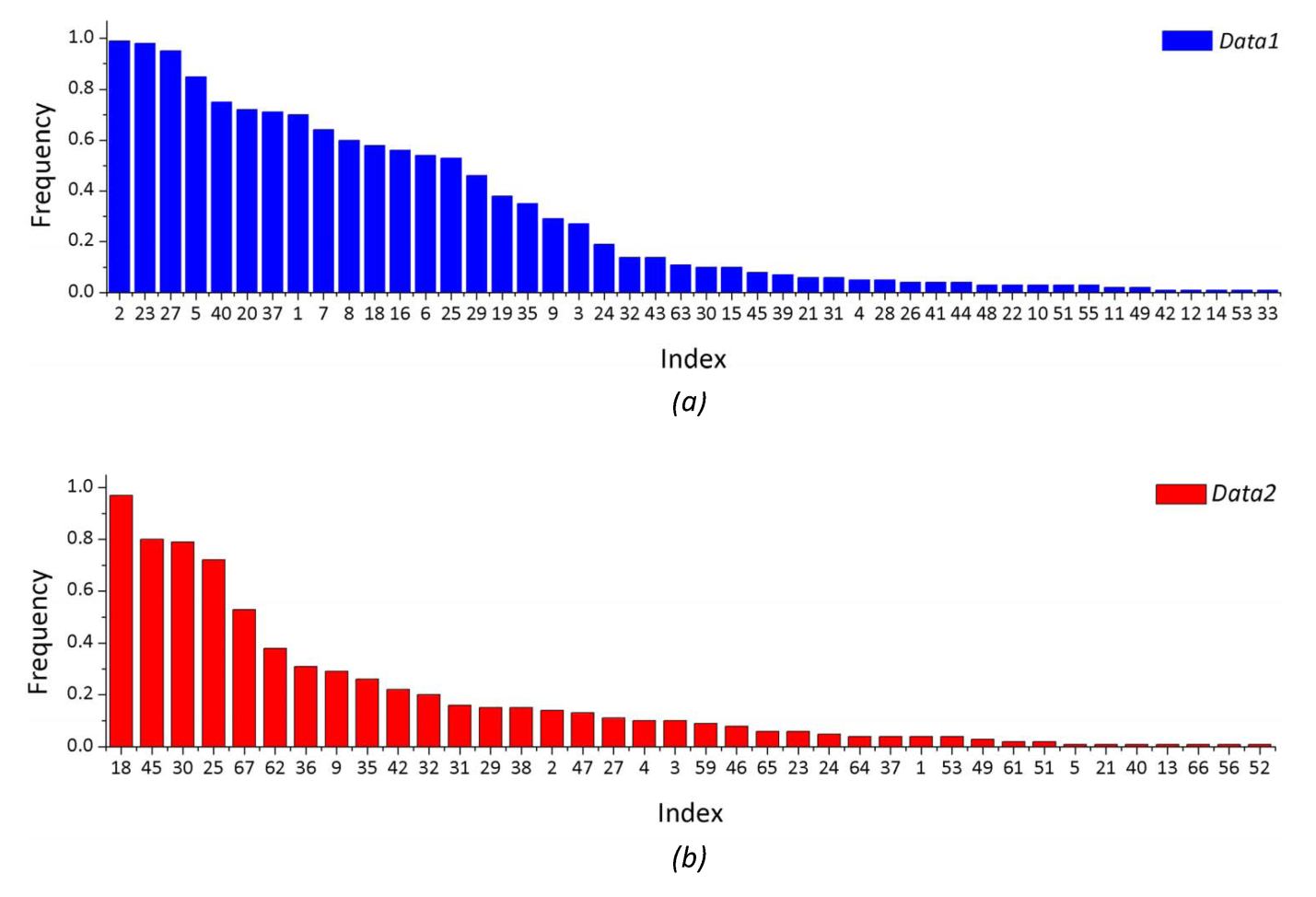


**Figure S2. Frequency of optimal features across cross-validation.**

The features in the optimal feature set are relatively constant for both datasets (a) *Data1* (b) *Data2* over all folds of cross-validation. The indices are corresponding to the indices in Figure 10.

# Tables

## Table S1. Registration results with simulated images.

| Varied Parameters | Registration Error (*s, θ,* *t_x_, t_y_*) |
| --- | --- |
| *s* | (0.0109, 0.4735, 0.7705, 0.6874) |
| *θ* | (0.0042, 0.4941, 0.0991, 0.0900) |
| *t_x_, t_y_* | (0.0028, 0.0662, 0.5068, 0.5734) |
| *s, θ, t_x_, t_y_* | (0.0097, 3.4416, 0.9626, 0.7353) |

## For each case, the average registration error in the recovered parameters is computed over 100 simulated images. Scaling *s*, rotation angle *θ*, and translation (*t_x_, t_y_*) errors are given relative to the original image scale, in degrees, and in pixels, respectively.

**Table S2 . Classification results varying parameter values via cross-validation.**

| $\gamma$ | Dataset | Feature Extraction | AUC | | Sensitivity (%) | Specificity (%) | | M_f_ |
| --- | --- | --- | --- | --- | --- | --- | --- | --- |
|  |  |  | AVG | STD |  | AVG | STD |  |
| 10 | *Data1* | IR & HE | 0.967 | 0.0059 | 90 | 88.40 | 3.74 | 9 |
|  |  |  |  |  | 95 | 80.77 | 5.77 |  |
|  |  |  |  |  | 99 | 62.47 | 6.51 |  |
|  |  | HE only | 0.945 | 0.0058 | 90 | 83.21 | 4.45 | 10 |
|  |  |  |  |  | 95 | 72.63 | 5.10 |  |
|  |  |  |  |  | 99 | 36.78 | 14.03 |  |
|  | *Data2* | IR & HE | 0.914 | 0.0208 | 90 | 63.14 | 11.26 | 4 |
|  |  |  |  |  | 95 | 42.43 | 9.71 |  |
|  |  |  |  |  | 99 | 31.34 | 8.92 |  |
|  |  | HE only | 0.735 | 0.0659 | 90 | 30.18 | 9.88 | 8 |
|  |  |  |  |  | 95 | 15.69 | 8.31 |  |
|  |  |  |  |  | 99 | 5.04 | 3.79 |  |
| 0.1 | *Data1* | IR & HE | 0.974 | 0.0048 | 90 | 93.98 | 2.18 | 17.5 |
|  |  |  |  |  | 95 | 86.91 | 3.82 |  |
|  |  |  |  |  | 99 | 68.08 | 8.29 |  |
|  |  | HE only | 0.959 | 0.0043 | 90 | 92.46 | 1.73 | 13 |
|  |  |  |  |  | 95 | 82.75 | 2.92 |  |
|  |  |  |  |  | 99 | 39.75 | 5.53 |  |
|  | *Data2* | IR & HE | 0.963 | 0.0174 | 90 | 90.48 | 9.46 | 8 |
|  |  |  |  |  | 95 | 80.40 | 15.38 |  |
|  |  |  |  |  | 99 | 39.59 | 22.07 |  |
|  |  | HE only | 0.901 | 0.0073 | 90 | 70.31 | 9.82 | 12 |
|  |  |  |  |  | 95 | 33.79 | 13.92 |  |
|  |  |  |  |  | 99 | 15.67 | 12.47 |  |
| 0.01 | *Data1* | IR & HE | 0.970 | 0.0053 | 90 | 93.47 | 1.66 | 13 |
|  |  |  |  |  | 95 | 85.50 | 5.77 |  |
|  |  |  |  |  | 99 | 51.31 | 13.05 |  |
|  |  | HE only | 0.955 | 0.0078 | 90 | 90.76 | 2.64 | 12 |
|  |  |  |  |  | 95 | 78.77 | 3.84 |  |
|  |  |  |  |  | 99 | 28.77 | 11.99 |  |
|  | *Data2* | IR & HE | 0.973 | 0.0160 | 90 | 93.44 | 5.57 | 9 |
|  |  |  |  |  | 95 | 84.44 | 6.64 |  |
|  |  |  |  |  | 99 | 49.46 | 28.54 |  |
|  |  | HE only | 0.894 | 0.0218 | 90 | 67.57 | 13.59 | 12 |
|  |  |  |  |  | 95 | 37.36 | 15.81 |  |
|  |  |  |  |  | 99 | 8.87 | 7.1 |  |
| 0.001 | *Data1* | IR & HE | 0.969 | 0.0074 | 90 | 92.70 | 3.04 | 12 |
|  |  |  |  |  | 95 | 83.47 | 5.42 |  |
|  |  |  |  |  | 99 | 51.80 | 15.65 |  |
|  |  | HE only | 0.954 | 0.0059 | 90 | 90.62 | 3.59 | 13 |
|  |  |  |  |  | 95 | 79.19 | 3.77 |  |
|  |  |  |  |  | 99 | 22.41 | 6.84 |  |
|  | *Data2* | IR & HE | 0.967 | 0.0139 | 90 | 92.24 | 3.46 | 10 |
|  |  |  |  |  | 95 | 85.07 | 6.14 |  |
|  |  |  |  |  | 99 | 40.84 | 24.25 |  |
|  |  | HE only | 0.879 | 0.0186 | 90 | 59.01 | 15.58 | 12 |
|  |  |  |  |  | 95 | 24.93 | 13.11 |  |
|  |  |  |  |  | 99 | 6.73 | 5.97 |  |

AVG and STD denote average and standard deviation across ten repeats of cross-valdiation. M_f_ is the median size of the feature set obtained by feature selection from training data. Column “Feature Extraction” indicates if features were obtained using H&E as well as IR data, or with H&E data alone. *γ* is the parameter of a radial basis kernel for SVM.

**Table S3. Classification results on *Data2* varying parameter values.**

| $\gamma$ | Feature Extraction | Dataset | AUC | | Sensitivity (%) | Specificity (%) | | M_f_ |
| --- | --- | --- | --- | --- | --- | --- | --- | --- |
|  |  |  | AVG | STD |  | AVG | STD |  |
| 10 | IR & HE | Train | 0.999 | 0.0010 | 90 | 100.00 | 0.00 | 10.5 |
|  |  |  |  |  | 95 | 99.80 | 0.78 |  |
|  |  |  |  |  | 99 | 97.90 | 2.44 |  |
|  |  | Test | 0.849 | 0.0401 | 90 | 63.60 | 12.20 |  |
|  |  |  |  |  | 95 | 46.90 | 17.55 |  |
|  |  |  |  |  | 99 | 24.29 | 6.99 |  |
|  | HE only | Train | 0.999 | 0.0003 | 90 | 100.00 | 0.00 | 10.5 |
|  |  |  |  |  | 95 | 99.85 | 0.33 |  |
|  |  |  |  |  | 99 | 98.84 | 0.90 |  |
|  |  | Test | 0.846 | 0.0442 | 90 | 41.76 | 13.47 |  |
|  |  |  |  |  | 95 | 28.16 | 12.89 |  |
|  |  |  |  |  | 99 | 13.66 | 9.39 |  |
| 0.1 | IR & HE | Train | 0.987 | 0.0004 | 90 | 96.13 | 0.60 | 38 |
|  |  |  |  |  | 95 | 93.77 | 0.76 |  |
|  |  |  |  |  | 99 | 86.11 | 1.39 |  |
|  |  | Test | 0.917 | 0.0069 | 90 | 70.68 | 1.99 |  |
|  |  |  |  |  | 95 | 59.71 | 3.55 |  |
|  |  |  |  |  | 99 | 28.29 | 3.69 |  |
|  | HE only | Train | 0.979 | 0.0018 | 90 | 97.50 | 1.56 | 14 |
|  |  |  |  |  | 95 | 91.95 | 2.90 |  |
|  |  |  |  |  | 99 | 52.41 | 13.10 |  |
|  |  | Test | 0.896 | 0.0135 | 90 | 51.90 | 5.89 |  |
|  |  |  |  |  | 95 | 32.46 | 8.99 |  |
|  |  |  |  |  | 99 | 3.16 | 1.85 |  |
| 0.01 | IR & HE | Train | 0.984 | 0.0031 | 90 | 96.08 | 1.01 | 34 |
|  |  |  |  |  | 95 | 94.36 | 2.73 |  |
|  |  |  |  |  | 99 | 80.70 | 6.82 |  |
|  |  | Test | 0.934 | 0.0052 | 90 | 76.48 | 2.62 |  |
|  |  |  |  |  | 95 | 64.29 | 3.01 |  |
|  |  |  |  |  | 99 | 32.57 | 3.01 |  |
|  | HE only | Train | 0.985 | 0.0225 | 90 | 97.98 | 4.44 | 15 |
|  |  |  |  |  | 95 | 90.44 | 14.87 |  |
|  |  |  |  |  | 99 | 87.23 | 16.68 |  |
|  |  | Test | 0.893 | 0.0143 | 90 | 53.13 | 17.10 |  |
|  |  |  |  |  | 95 | 25.76 | 6.63 |  |
|  |  |  |  |  | 99 | 8.11 | 5.36 |  |
| 0.001 | IR & HE | Train | 0.984 | 0.0032 | 90 | 96.06 | 0.95 | 45 |
|  |  |  |  |  | 95 | 94.00 | 1.95 |  |
|  |  |  |  |  | 99 | 78.84 | 6.16 |  |
|  |  | Test | 0.937 | 0.0105 | 90 | 78.09 | 5.62 |  |
|  |  |  |  |  | 95 | 65.00 | 6.78 |  |
|  |  |  |  |  | 99 | 32.57 | 3.01 |  |
|  | HE only | Train | 0.977 | 0.0290 | 90 | 93.85 | 11.40 | 13.5 |
|  |  |  |  |  | 95 | 83.45 | 24.27 |  |
|  |  |  |  |  | 99 | 81.64 | 27.07 |  |
|  |  | Test | 0.895 | 0.0141 | 90 | 58.81 | 9.71 |  |
|  |  |  |  |  | 95 | 26.07 | 10.47 |  |
|  |  |  |  |  | 99 | 9.33 | 4.58 |  |

A classifier is trained on *Data1* and tested on *Data2*. AVG and STD denote the average and standard deviation. M_f_ is the median size of the optimal feature set. Column “Feature Extraction” indicates if features were obtained using H&E as well as IR data, or with H&E data alone. Column “Dataset” indicates if the performance metrics are from training data (*Data1*) or from test data (*Data2*). *γ* is the parameter of a radial basis kernel for SVM.

**Table S4. Classification results on *Data1* varying parameter values.**

| $\gamma$ | Feature Extraction | Dataset | AUC | | Sensitivity (%) | Specificity (%) | | M_f_ |
| --- | --- | --- | --- | --- | --- | --- | --- | --- |
|  |  |  | AVG | STD |  | AVG | STD |  |
| 1 | IR & HE | Train | 0.998 | 0.0007 | 90 | 100.00 | 0.00 | 9 |
|  |  |  |  |  | 95 | 99.71 | 0.37 |  |
|  |  |  |  |  | 99 | 95.37 | 1.75 |  |
|  |  | Test | 0.855 | 0.0340 | 90 | 50.18 | 11.52 |  |
|  |  |  |  |  | 95 | 40.41 | 9.88 |  |
|  |  |  |  |  | 99 | 12.33 | 6.29 |  |
|  | HE only | Train | 0.997 | 0.0050 | 90 | 100.00 | 0.00 | 8 |
|  |  |  |  |  | 95 | 95.36 | 7.91 |  |
|  |  |  |  |  | 99 | 92.79 | 10.20 |  |
|  |  | Test | 0.804 | 0.0427 | 90 | 48.58 | 10.16 |  |
|  |  |  |  |  | 95 | 37.75 | 9.96 |  |
|  |  |  |  |  | 99 | 22.03 | 9.72 |  |
| 10 | IR & HE | Train | 0.998 | 0.0018 | 90 | 99.80 | 0.63 | 7 |
|  |  |  |  |  | 95 | 99.26 | 1.62 |  |
|  |  |  |  |  | 99 | 97.08 | 4.17 |  |
|  |  | Test | 0.719 | 0.0782 | 90 | 29.41 | 19.22 |  |
|  |  |  |  |  | 95 | 21.83 | 19.42 |  |
|  |  |  |  |  | 99 | 8.12 | 11.02 |  |
|  | HE only | Train | 0.998 | 0.0018 | 90 | 99.80 | 0.63 | 10 |
|  |  |  |  |  | 95 | 99.26 | 1.62 |  |
|  |  |  |  |  | 99 | 97.08 | 4.17 |  |
|  |  | Test | 0.773 | 0.0534 | 90 | 39.99 | 10.12 |  |
|  |  |  |  |  | 95 | 24.71 | 11.45 |  |
|  |  |  |  |  | 99 | 12.35 | 7.74 |  |
| 0.1 | IR & HE | Train | 0.999 | 0.0009 | 90 | 100.00 | 0.00 | 11.5 |
|  |  |  |  |  | 95 | 99.71 | 0.90 |  |
|  |  |  |  |  | 99 | 97.09 | 2.56 |  |
|  |  | Test | 0.839 | 0.0287 | 90 | 39.90 | 11.64 |  |
|  |  |  |  |  | 95 | 29.96 | 9.67 |  |
|  |  |  |  |  | 99 | 7.94 | 4.77 |  |
|  | HE only | Train | 0.988 | 0.0053 | 90 | 100.00 | 0.00 | 9 |
|  |  |  |  |  | 95 | 93.00 | 5.96 |  |
|  |  |  |  |  | 99 | 69.00 | 12.62 |  |
|  |  | Test | 0.768 | 0.0426 | 90 | 33.06 | 13.28 |  |
|  |  |  |  |  | 95 | 20.51 | 11.69 |  |
|  |  |  |  |  | 99 | 5.05 | 4.79 |  |
| 0.01 | IR & HE | Train | 0.999 | 0.0011 | 90 | 100.00 | 0.00 | 13 |
|  |  |  |  |  | 95 | 99.64 | 0.91 |  |
|  |  |  |  |  | 99 | 97.87 | 2.79 |  |
|  |  | Test | 0.840 | 0.0332 | 90 | 39.77 | 12.94 |  |
|  |  |  |  |  | 95 | 27.99 | 10.46 |  |
|  |  |  |  |  | 99 | 6.02 | 3.08 |  |
|  | HE only | Train | 0.988 | 0.0042 | 90 | 100.00 | 0.00 | 11 |
|  |  |  |  |  | 95 | 91.43 | 8.42 |  |
|  |  |  |  |  | 99 | 68.74 | 10.99 |  |
|  |  | Test | 0.773 | 0.0528 | 90 | 30.62 | 16.98 |  |
|  |  |  |  |  | 95 | 15.11 | 13.58 |  |
|  |  |  |  |  | 99 | 2.28 | 2.87 |  |
| 0.001 | IR & HE | Train | 0.999 | 0.0011 | 90 | 100.00 | 0.00 | 13 |
|  |  |  |  |  | 95 | 99.64 | 0.91 |  |
|  |  |  |  |  | 99 | 97.87 | 2.79 |  |
|  |  | Test | 0.837 | 0.0240 | 90 | 39.46 | 9.58 |  |
|  |  |  |  |  | 95 | 24.86 | 5.27 |  |
|  |  |  |  |  | 99 | 6.59 | 3.93 |  |
|  | HE only | Train | 0.984 | 0.0066 | 90 | 97.38 | 5.56 | 11 |
|  |  |  |  |  | 95 | 84.21 | 11.51 |  |
|  |  |  |  |  | 99 | 66.57 | 4.80 |  |
|  |  | Test | 0.769 | 0.0417 | 90 | 29.31 | 13.71 |  |
|  |  |  |  |  | 95 | 14.03 | 8.25 |  |
|  |  |  |  |  | 99 | 3.83 | 5.65 |  |

A classifier is trained on *Data2* and tested on *Data1*. AVG and STD denote the average and standard deviation. M_f_ is the median size of the optimal feature set. Column “Feature Extraction” indicates if features were obtained using H&E as well as IR data, or with H&E data alone. Column “Dataset” indicates if the performance metrics are from training data (*Data2*) or from test data (*Data1*). *γ* is the parameter of a radial basis kernel for SVM.
